# Antibiotic Capture by Bacterial Lipocalins Uncovers an Extracellular Mechanism of Intrinsic Antibiotic Resistance

**DOI:** 10.1128/mBio.00225-17

**Published:** 2017-03-14

**Authors:** Omar M. El-Halfawy, Javier Klett, Rebecca J. Ingram, Slade A. Loutet, Michael E. P. Murphy, Sonsoles Martín-Santamaría, Miguel A. Valvano

**Affiliations:** a Department of Microbiology and Immunology, University of Western Ontario, London, Ontario, Canada; b Microbiology and Immunology Department, Faculty of Pharmacy, Alexandria University, Alexandria, Egypt; c Department of Chemical and Physical Biology, Centre for Biological Research, CIB, CSIC, Madrid, Spain; d The Wellcome-Wolfson Institute of Experimental Medicine, Queen’s University Belfast, Belfast, United Kingdom; e Department of Microbiology and Immunology, University of British Columbia, Vancouver, British Columbia, Canada; Emory University School of Medicine

## Abstract

The potential for microbes to overcome antibiotics of different classes before they reach bacterial cells is largely unexplored. Here we show that a soluble bacterial lipocalin produced by *Burkholderia cenocepacia* upon exposure to sublethal antibiotic concentrations increases resistance to diverse antibiotics *in vitro* and *in vivo*. These phenotypes were recapitulated by heterologous expression in *B. cenocepacia* of lipocalin genes from *Pseudomonas aeruginosa*, *Mycobacterium tuberculosis*, and methicillin-resistant *Staphylococcus aureus*. Purified lipocalin bound different classes of bactericidal antibiotics and contributed to bacterial survival *in vivo*. Experimental and X-ray crystal structure-guided computational studies revealed that lipocalins counteract antibiotic action by capturing antibiotics in the extracellular space. We also demonstrated that fat-soluble vitamins prevent antibiotic capture by binding bacterial lipocalin with higher affinity than antibiotics. Therefore, bacterial lipocalins contribute to antimicrobial resistance by capturing diverse antibiotics in the extracellular space at the site of infection, which can be counteracted by known vitamins.

## IMPORTANCE

Current research on antibiotic action and resistance focuses on targeting essential functions within bacterial cells. We discovered a previously unrecognized mode of general bacterial antibiotic resistance operating in the extracellular space, which depends on bacterial protein molecules called lipocalins. These molecules are highly conserved in most bacteria and have the ability to capture different classes of antibiotics outside bacterial cells. We also discovered that liposoluble vitamins, such as vitamin E, overcome *in vitro* and *in vivo* antibiotic resistance mediated by bacterial lipocalins, providing an unexpected new alternative to combat resistance by using this vitamin or its derivatives as antibiotic adjuvants.

## INTRODUCTION

Treating infections is becoming increasingly difficult since microbes often show intrinsic high-level resistance to virtually all clinically approved antibiotics (1). Ineffective microbial killing and exposure to sublethal antibiotic concentrations elicit adaptive bacterial stress responses, enhancing antibiotic resistance and tolerance (2–7). Much has been learned about antibiotic resistance mechanisms at the cellular level (8, 9), but whether microbes subvert the action of antibiotics before they come in contact with bacterial cells has remained largely unexplored, with the exception of β-lactamases, which are often trapped in released membrane vesicles (10–13), and the recent observation that *Staphylococcus aureus* inactivates daptomycin by releasing membrane phospholipids (14).

*Burkholderia cenocepacia* is a highly multidrug resistant, opportunistic, Gram-negative bacterium that causes serious respiratory infections in patients with cystic fibrosis (15). Bacteria of the genus *Burkholderia* are notorious for their ability to resist the action of multiple classes of antimicrobials (16), representing an attractive model to understand intrinsic mechanisms of resistance in opportunistic bacteria. Recently, we showed that in response to sublethal antibiotic concentrations, *B. cenocepacia* produces and releases molecules such as the polyamine putrescine and YceI, a conserved hypothetical protein of unknown function (17). YceI proteins are a family of bacterial lipocalins (BCNs), which are small proteins widely conserved in Gram-negative and Gram-positive bacteria but whose physiological role is unclear (18, 19). In coculture experiments, *B. cenocepacia* protects *Pseudomonas aeruginosa* from killing by different bactericidal antibiotics (17). This effect was abrogated in the *B. cenocepacia* Δ*bcnA-bcnB* double-deletion mutant (17), but the individual contribution of each BCN paralog and its mechanism remained unknown.

Here, we show that secreted BcnA contributes to increased resistance of *B. cenocepacia* to various classes of antibiotics *in vitro* and *in vivo*. The expression of BCN orthologs from *P. aeruginosa*, *Mycobacterium tuberculosis*, and methicillin-resistant *S. aureus* in a *B. cenocepacia* Δ*bcnA* mutant recapitulated this function. Experimental and computational studies revealed that BCNs bind to a range of antibiotics, thus preventing their antibacterial activity and contributing to resistance. X-ray crystallography studies of BCN structures, in combination with docking and MD simulations, have helped us to rationalize plausible binding modes. We also discovered that fat-soluble vitamins bound BcnA with a higher affinity than antibiotics, enabling them to outcompete antibiotics. This finding provides a clinically applicable strategy whereby known vitamins could become antibiotic adjuvants by increasing the concentration of free antibiotics in the proximity of bacterial cells, thereby boosting their microbicidal activity.

## RESULTS

### BcnA is a secreted bacterial lipocalin required for full resistance of *B. cenocepacia* to different classes of antibiotics.

**FIG 1:**
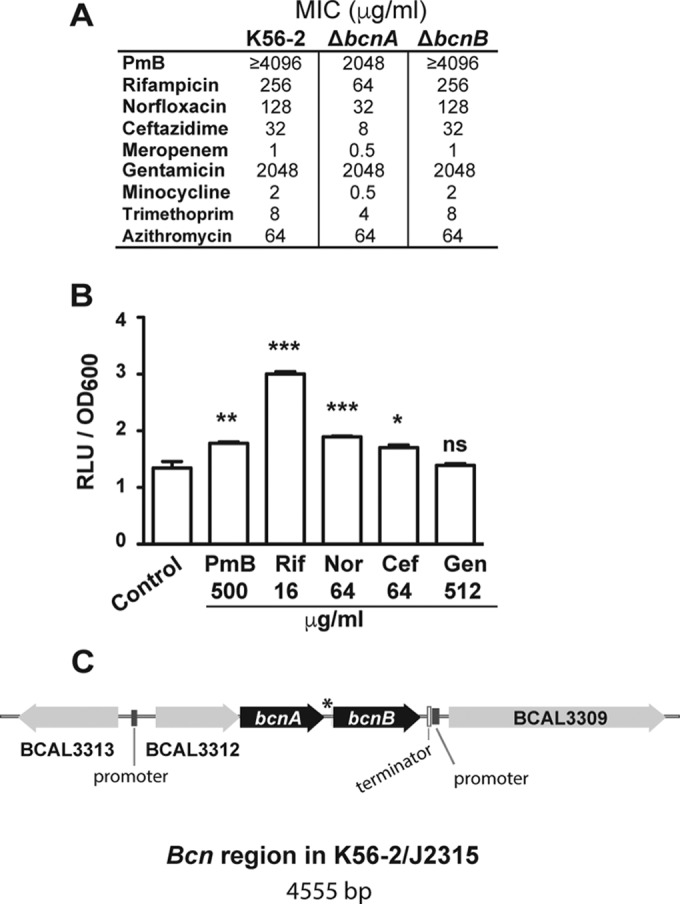
*B. cenocepacia* BcnA confers resistance to hydrophobic but not hydrophilic antibiotics. (A) MICs of different antibiotics determined by broth microdilution in cation-adjusted MHB at 18 to 24 h (representative of three independent experiments). (B) Luciferase expression assay of *B. cenocepacia* BcnA (OME61) in response to antibiotics at 3 h (*n* = 6 from two different clones). Results are shown as the mean number of relative light units (RLU)/OD_600_ unit ± SEM. The percent OD_600_ values are shown in Fig. S2D. *, *P* < 0.05; **, *P* < 0.01; ***, *P* < 0.001 (from one-way ANOVA [overall *P* < 0.001] and the Bonferroni *post hoc* test comparing against the non-antibiotic-treated control conditions). At the chosen sample size, the actual power of the assay to detect significant effects at a significance level (alpha) of 0.05, two tailed, is >99% in the case of PmB and Rif and 90% for the rest of the conditions. ns, not significant. (C) Genomic organization of the *B. cenocepacia* K56-2 region. The asterisk indicates that the transcribed intergenic sequence between *bcnA* and *bcnB* has the potential to form strong secondary structures, as determined with Mfold (http://unafold.rna.albany.edu/?q=mfold/download-mfold) (71).

We investigated the role of *B. cenocepacia* BcnA (BCAL3311) and BcnB (BCAL3310) by constructing individual deletion mutants in their corresponding genes and assessing bacterial susceptibility to model bactericidal antibiotics representing different classes, including rifamycins (mRNA transcription inhibitors), fluoroquinolones (DNA replication inhibitors), several β-lactams (cell wall peptidoglycan synthesis inhibitors), and cationic antimicrobial peptides (cell membrane active agents). The Δ*bcnA* mutant, but not the Δ*bcnB* mutant, had increased susceptibility (4-fold MIC reduction) to rifampin, norfloxacin, ceftazidime, and the cationic antimicrobial peptide polymyxin B (PmB) and a 2-fold meropenem MIC reduction. No effect was observed with the aminoglycoside gentamicin (protein synthesis inhibitor) (Fig. 1A; see antibiotic chemical structures in Fig. S1 in the supplemental material). Similarly, we also tested model bacteriostatic antibiotics representing different classes. The Δ*bcnA* mutant, but not the Δ*bcnB* mutant, had increased susceptibility to minocycline (tetracycline family protein synthesis inhibitor; 4-fold MIC reduction) and trimethoprim (pyrimidine inhibitor of bacterial dihydrofolate reductase; 2-fold MIC reduction), while no effect was observed with the macrolide (protein synthesis inhibitor) azithromycin (Fig. 1A; see Fig. S1).

10.1128/mBio.00225-17.2 FIG S1  Chemical structures of the antibiotics and chemicals used in this study. Download FIG S1, PDF file, 0.2 MB. Copyright © 2017 El-Halfawy et al. 2017 El-Halfawy et al. This content is distributed under the terms of the Creative Commons Attribution 4.0 International license.

The expression of the *bcnA* and *bcnB* genes in response to antibiotics at concentrations near the MIC (sublethal) was characterized by constructing chromosomal *lux* fusions. Expression of *bcnA*::*luxCDABE* was upregulated upon exposure to PmB, rifampin, norfloxacin, and ceftazidime (Fig. 1B). In contrast, *bcnA*::*luxCDABE* expression in response to gentamicin remained unchanged (Fig. 1B). Expression of *bcnB*::*luxCDABE* only increased upon exposure to norfloxacin and ceftazidime (Fig. S2A). Thus, the *bcnA* and *bcnB* genes respond to antibiotic stress, but they appeared to be differentially regulated. *bcnA* and *bcnB* are located on the same strand 63 bp apart (Fig. 1C). Immediately upstream of *bcnA*, there is an open reading frame (BCAL3312), also transcribed on the same strand, that encodes a predicted cytochrome *b*_651_ protein. Putative promoter regions are found upstream of BCAL3312 and downstream from *bcnB*, next to a predicted Rho-independent transcription termination sequence. The genomic organization of the *bcn* region suggests that BCAL3312, *bcnA*, and *bcnB* are cotranscribed and may form an operon. However, the transcribed 63-bp intergenic region between *bcnA* and *bcnB* has the potential to form strong RNA secondary structures, which might explain the differential regulation of the two genes by antibiotics. The secretion of the BcnA and BcnB proteins was also investigated by using FLAG-tagged derivatives; only BcnA was secreted extracellularly into the growth medium (Fig. S2B). From these experiments, we concluded that BcnA is the major contributor to intrinsic antibiotic resistance upon antibiotic stress and is secreted into the extracellular bacterial milieu.

10.1128/mBio.00225-17.3 FIG S2  Expression and secretion profiles of BcnA and BcnB. Download FIG S2, PDF file, 0.2 MB. Copyright © 2017 El-Halfawy et al. 2017 El-Halfawy et al. This content is distributed under the terms of the Creative Commons Attribution 4.0 International license.

### BCN orthologs from different species restore BcnA function in *B. cenocepacia*.

**FIG 2:**
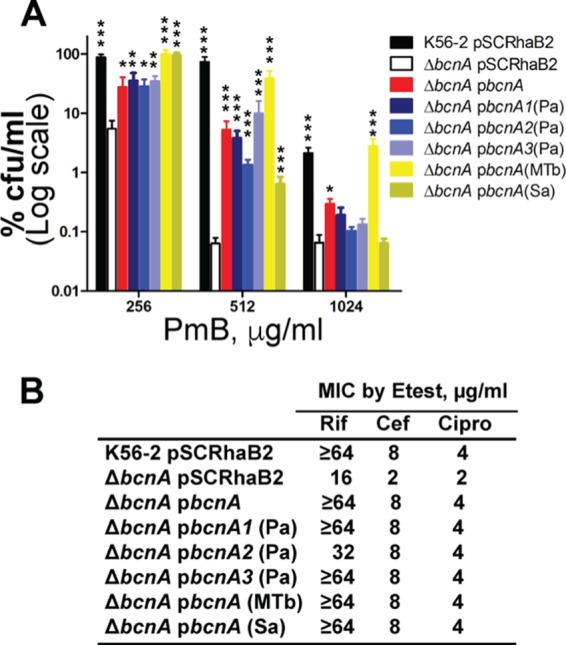
BCNs from different bacteria can restore the *B. cenocepacia* Δ*bcnA* mutant to full antibiotic resistance. (A) Mean CFU count ± SEM on LB agar containing PmB from three independent experiments (*n* = 6; asterisks denote differences from the Δ*bcnA*/pSCRhaB2 mutant). *, *P* < 0.05; **, *P* < 0.01; ***, *P* < 0.001 (determined by two-way ANOVA and the Bonferroni *post hoc* test on the log CFU-per-milliliter values with an overall *P* value of <0.001 for variations across the different mutants and because of different concentrations). At the chosen sample size, the actual power of the assay to detect statistically significant effects at a significance level (alpha) of 0.05, two tailed, ranged between 85 and >99% for the different mutants tested. (B) MICs of rifampin (Rif), ceftazidime (Cef), and ciprofloxacin (Cipro) determined by E test in a representative of three independent experiments. The highest rifampin concentration on the E-test strips is 32 µg/ml; ≥64 indicates that an MIC could not be detected within the E-test concentration range and would be ≥64 µg/ml.

To demonstrate whether BCN orthologs from other bacteria could restore BcnA function in the Δ*bcnA* mutant strain, we tested BCNs of *P. aeruginosa* PAO1 [PA0423, PA4340, and PA4345, which are BcnA1(Pa), BcnA2(Pa), and BcnA3(Pa), respectively, here], *M. tuberculosis* H37Rv [Rv1890c, which is BcnA(MTb) here], and community-acquired methicillin-resistant *S. aureus* USA300 [SAUSA300_2620, which is BcnA(Sa) here]. CFU counts on PmB-containing plates (Fig. 2A) and E-test MICs of rifampin, ceftazidime, and ciprofloxacin (a fluoroquinolone closely related to norfloxacin) (Fig. 2B) showed that heterologous expression of BCNs from these different bacteria restores antibiotic resistance to parental levels, indicating that these proteins have a conserved function.

### BcnA sequesters antibiotics.

**FIG 3:**
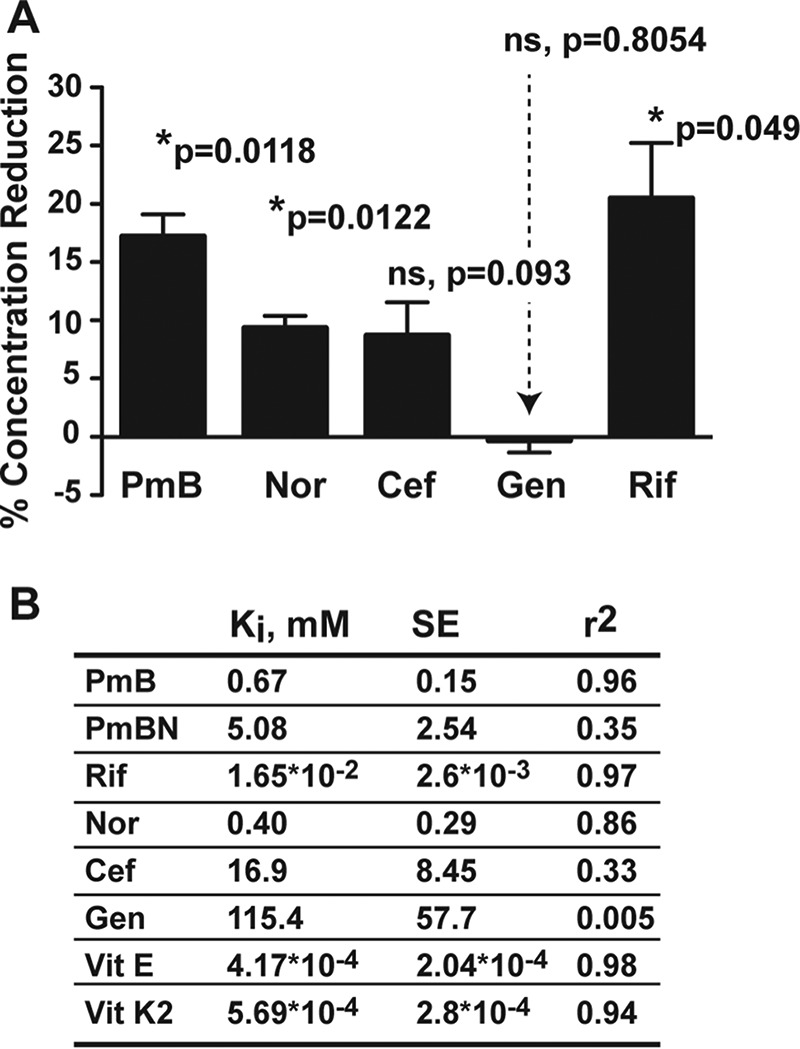
BCNs bind antibiotics and other molecules hydrophobic in nature with high affinities. (A) Antibiotic assay showing the mean reduction in the concentration of hydrophobic antibiotics because of sequestration by BcnA ± SEM (*n* = 3 from three independent experiments). *, *P* < 0.05 (determined by one-sample *t* test compared to a hypothetical 0% antibiotic sequestration). At the chosen sample size, the actual power of the assay to detect statistically significant effects at a significance level (alpha) of 0.05, two tailed, is >99%. ns, not significant. (B) *In vitro* binding assay showing mean binding inhibition constants (*K*_*i*_ values) of antibiotics against 1.5 µM Nile red binding to 1.5 µM BcnA in PBS in three independent experiments (*n* = 5).

BCNs bind to diverse hydrophobic molecules (20–22); hence, we hypothesized that BCNs could capture antibiotics and reduce their effective concentration in the bacterial milieu. An antibiotic bioassay demonstrated that BcnA sequestered rifampin, PmB, norfloxacin, and ceftazidime, in descending order of magnitude, but not gentamicin (Fig. 3A; see Fig. S3A to E). Further, the relative affinity of BcnA for antibiotics was determined *in vitro* by binding competition of antibiotics with Nile red in complex with BcnA. Nile red is a fluorophore used to test hydrophobic binding sites in proteins (23). The calculated binding inhibitory constants (*K*_*i*_ values) of the antibiotics (Fig. 3B; see Fig. S3F to M) mirrored their relative abilities to be sequestered by BcnA (Fig. 3A) and the antibiotic susceptibility phenotypes of the Δ*bcnA* mutant (Fig. 1A). Notably, the binding of BcnB to Nile red occurred with ~20-fold lower affinity than that of BcnA (Fig. S3N to Q), agreeing with the lesser role of BcnB in antibiotic resistance. The involvement of hydrophobic moieties in the interaction of ligands with BcnA was suggested by the significantly higher *K*_*i*_ value of the PmB nonapeptide, which lacks the hydrophobic N-terminal tail of PmB (24), relative to that of PmB (Fig. 3B; see Fig. S3F to I). Notably, azithromycin and gentamicin, the only antibiotics not showing the antibiotic sensitivity reduction phenotype with the Δ*bcnA* mutant (Fig. 1A), are the only antibiotics tested that lack aromatic or hydrophobic moieties (Fig. S1). To test polar hydrophilic binding sites in BcnA compared to hydrophobic sites, we used two related BODIPY dye-labeled phospholipids. BcnA increased the intensity of fluorescence of the BODIPY fluorophore when attached to the fatty acyl chain of the phospholipid in BODIPY-phosphocholine but not when attached to the hydrophilic polar head group (BODIPY-phosphoethanolamine) (Fig. S3R to T). This further supported a role for fatty acyl chains in the interaction with hydrophobic sites in BcnA.

10.1128/mBio.00225-17.4 FIG S3  Binding assay results. Download FIG S3, PDF file, 0.4 MB. Copyright © 2017 El-Halfawy et al. 2017 El-Halfawy et al. This content is distributed under the terms of the Creative Commons Attribution 4.0 International license.

### Structure determination and molecular modeling reveal distinct docked binding modes for BcnA.

**FIG 4:**
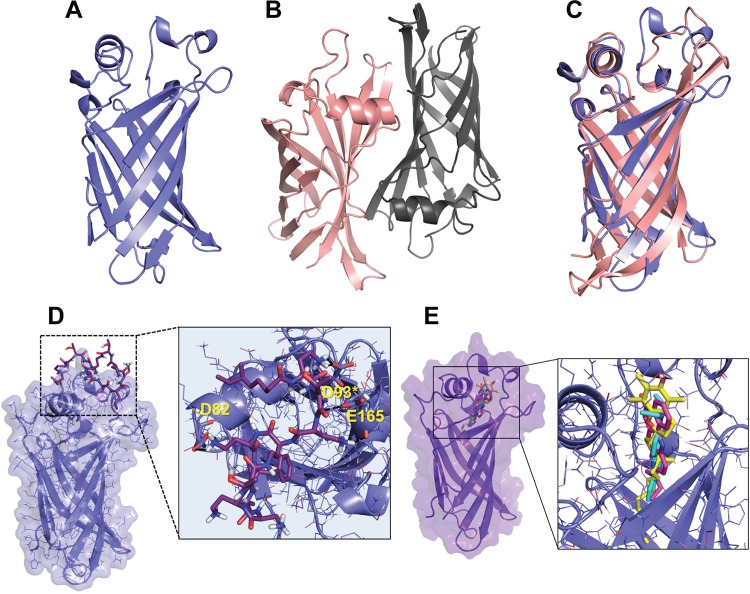
Structural analysis of BcnA and BcnB and ligand docking modeling. (A) BcnA (blue) is a monomer. (B) BcnB (protomer 1, chain C, peach; protomer 2, chain D, gray) is a dimer. (C) Superposition of BcnA and BcnB with dissimilar tunnel opening loops boxed. (D) Docked structure of PmB in complex with the BcnA crystallographic structure. (E) Docking model presenting the superimposition of the two best predicted binding modes of Nile red (magenta and cyan) and α-tocopherol (yellow) when docked into the BcnA crystallographic structure.

To elucidate the mode of binding of antibiotics within BCNs, we first solved the X-ray crystal structures of BcnA and BcnB to 1.4- and 1.6-Å resolutions, respectively (Text S1, Table S1, and Fig. S4A to E). Visual inspection and structural alignments by the DALI server (25) confirmed a barrel-shaped lipocalin fold for both proteins (Fig. 4A and B). An octaprenyl pyrophosphate was bound within a long, hydrophobic tunnel extending from one end of the barrel in each structure (Fig. S4A and B). The binding of octaprenyl pyrophosphate potentially occurred during recombinant expression in *Escherichia coli*. Analysis with the PDBePISA server (26) predicted that BcnA is a monomer, while BcnB is a dimer, by crystallographic symmetry (~2,840 Å^2^ of buried surface area including a portion of the tunnel opening). These oligomeric states were confirmed in solution by size exclusion chromatography-multiangle light scattering (SEC-MALS; see Fig. S4C and D). Superposition of BcnA chain A and BcnB chain C (148 Cα atoms, 22% sequence identity) resulted in a root mean square deviation (RMSD) of 1.66 Å (Fig. 4C). The largest structural differences observed were located in two of the loops that make up the tunnel opening (Fig. 4C). These differences may play a role in the antibiotic binding potential of BcnA and BcnB.

10.1128/mBio.00225-17.1 TEXT S1  Supplemental results of this study. Download TEXT S1, PDF file, 0.1 MB. Copyright © 2017 El-Halfawy et al. 2017 El-Halfawy et al. This content is distributed under the terms of the Creative Commons Attribution 4.0 International license.

10.1128/mBio.00225-17.5 FIG S4  BcnA and BcnB macromolecular structures and models of antibiotic docking into and binding of the BcnA structure. Download FIG S4, PDF file, 1 MB. Copyright © 2017 El-Halfawy et al. 2017 El-Halfawy et al. This content is distributed under the terms of the Creative Commons Attribution 4.0 International license.

10.1128/mBio.00225-17.9 TABLE S1  X-ray crystallography data collection and refinement statistics. Download TABLE S1, PDF file, 0.1 MB. Copyright © 2017 El-Halfawy et al. 2017 El-Halfawy et al. This content is distributed under the terms of the Creative Commons Attribution 4.0 International license.

Using the established crystallographic structures, we applied molecular modeling and docking calculations to predict BCN binding modes of antibiotics (Text S1). These studies suggested two distinct docked binding modes for BcnA. One binding mode involved residues in the rim of the lipocalin pocket (Fig. 4D; see Fig. S4F to I). Polar interactions, mainly with polar residues, were observed with all of the antibiotics tested. There were also interactions between the aromatic moieties in PmB, rifampin, norfloxacin, and ceftazidime and lipophilic residues. These interactions were not observed with gentamicin, which lacks aromatic moieties and binds weakly to BcnA. The second binding mode was predicted for more lipophilic molecules (e.g., Nile red), occurring deeper inside the lipophilic tunnel (Fig. 4E). Further, analyses from molecular dynamic (MD) simulations suggested both structural and ligand recognition roles for residues D82 and D93 (Text S1). These residues are located in the loops at the top of the tunnel opening of BcnA. Site-directed mutagenesis was performed, and the BcnA_D82A-D93A_ mutant showed decreased binding affinity for Nile red (Fig. S5A and B), which we propose is due to structural changes (Text S1; Fig. S6A to E and S7). Docking of Nile red into the BcnA_D82A-D93A_ mutant (following the general docking protocol with the minimized average structure from ns 2.5 to ns 5 of the MD simulation) did not lead to any binding position inside the lipophilic tunnel (Fig. S7). Interestingly, D93 is a highly conserved residue in the consensus motif of the BCN protein family (Fig. S5C), exemplified in the alignment of BCN homologues (Fig. S5D). Further, Asp is found in positions equivalent to D82 in homologues of *B. cenocepacia* BcnA. Thus, it is credible that the mode of interaction between BcnA and antibiotics is common among this large family of conserved bacterial proteins.

10.1128/mBio.00225-17.6 FIG S5  Nile red binding affinities of *B. cenocepacia* BcnA site-directed mutants. Download FIG S5, PDF file, 0.4 MB. Copyright © 2017 El-Halfawy et al. 2017 El-Halfawy et al. This content is distributed under the terms of the Creative Commons Attribution 4.0 International license.

10.1128/mBio.00225-17.7 FIG S6  Overall MD simulation analysis of the BcnA, D82A-D93A mutant BcnA, and BcnB structures. Download FIG S6, PDF file, 0.5 MB. Copyright © 2017 El-Halfawy et al. 2017 El-Halfawy et al. This content is distributed under the terms of the Creative Commons Attribution 4.0 International license.

10.1128/mBio.00225-17.8 FIG S7  Effects of *B. cenocepacia* BCNs on different bacterial species *in vitro* and *in vivo*. Download FIG S7, PDF file, 0.2 MB. Copyright © 2017 El-Halfawy et al. 2017 El-Halfawy et al. This content is distributed under the terms of the Creative Commons Attribution 4.0 International license.

### Exogenous BcnA protects different bacterial species from antibiotic killing *in vitro* and *in vivo*.

**FIG 5:**
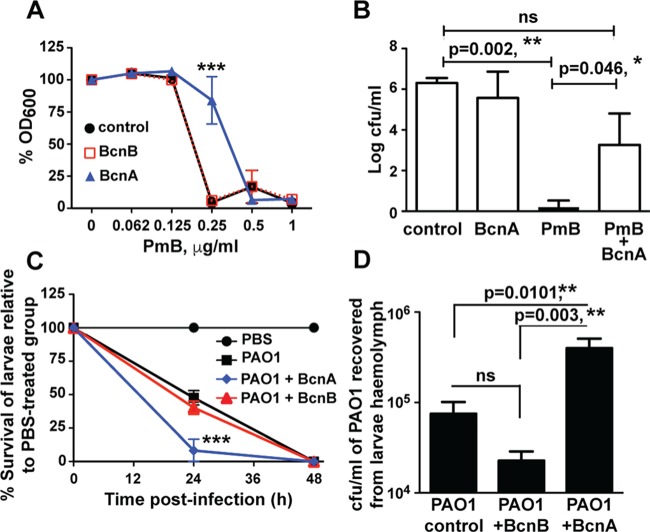
*B. cenocepacia* BcnA protects *P. aeruginosa in vitro* and *in vivo*. (A) *In vitro* protection of *P. aeruginosa* PAO1 against PmB with 1.5 µM BcnA or BcnB (*n* = 8 from four independent experiments, plotted as the mean ± SEM). PAO1 treated with BcnA is significantly different from control PAO1 and PAO1 plus BcnB at 0.25 µg/ml, as determined by two-way ANOVA with an overall *P* value of <0.001 and the Bonferroni *post hoc* test. At the chosen sample size, the actual power of the assay to detect statistically significant effects at a significance level (alpha) of 0.05, two tailed, ranged between 95 and 99%. (B) Protection of *P. aeruginosa* Q502 from PmB killing in an i.p. infection of C57BL/6 mice, plotted as the median plus the interquartile range. Significant differences were determined by the Kruskal-Wallis test with *P* = 0.001 and Dunn’s multiple-comparison test. At *n* = 6, the actual power of the assay to detect statistically significant effects at a significance level (alpha) of 0.05, two tailed, ranged between control and PmB-treated groups is >99%, and that between PmB and PmB plus BcnA-treated groups is 80%. ns, not significant. (C) The survival of *G. mellonella* larvae infected with *P. aeruginosa* PAO1 compared to control group injected with sterile PBS; 10 larvae/group; the results were obtained from three independent experiments and are shown as the mean ± SEM. The survival at 24 h of both PAO1- and PAO1-BcnB-treated larvae is significantly different from that of the PAO1-BcnA-treated group at *P* < 0.001 (determined by two-way ANOVA with an overall *P* value of <0.001 and the Bonferroni *post hoc* test). At the chosen sample size, the actual power of the assay to detect statistically significant effects at a significance level (alpha) of 0.05, two tailed, is 90%. (D) Numbers of PAO1 CFU per milliliter recovered from larval hemolymph at 200 min postinfection (*n* = 10 from two independent experiments shown as the mean ± SEM). **, *P* < 0.01 (one-way ANOVA with an overall *P* value of 0.006 and the Bonferroni *post hoc* test). At the chosen sample size, the actual power of the assay to detect statistically significant effects at a significance level (alpha) of 0.05, two tailed, is >99% in the case of BcnA versus BcnB-treated PAO1 and 85% in the case of BcnA-treated versus control PAO1.

Since *B. cenocepacia* BcnA is secreted (Fig. S2D), as predicted for most other BCNs, we hypothesized that exogenous BCNs produced by one bacterial species have the potential to protect other bacteria from the action of antibiotics, including antimicrobial peptides. This was investigated by using purified recombinant BCNs from *B. cenocepacia*. *In vitro* antibiotic protection assays showed that *P. aeruginosa* PAO1 treated with 1.5 µM purified BcnA, but not BcnB, had reduced sensitivity to PmB (Fig. 5A). In contrast, at 8- to 16-fold lower PmB concentrations, both proteins protected *Salmonella enterica* Typhi, *Shigella flexneri*, *Acinetobacter baumannii*, *Acinetobacter lwoffii*, and *Acinetobacter junii* strains (Fig. S7A). This disparity in the PmB concentrations at which protection by BcnA and BcnB occurs correlates with their relative affinities to Nile red (Fig. S3N to Q; see above). *In vivo* infections of C57BL/6 mice demonstrated that BcnA protected *P. aeruginosa* Q502, a virulent cystic fibrosis clinical isolate (27), from PmB killing in an intraperitoneal (i.p.) sepsis model (Fig. 5B). We also employed the *Galleria mellonella* larval infection model. The Δ*bcnA* and Δ*bcnB* mutants had reduced virulence in *G. mellonella* relative to that of the parent strain (Fig. S7B). However, significantly lower numbers of Δ*bcnA* mutant bacteria than parental and Δ*bcnB* mutant strain bacteria were recovered from the hemolymph of infected larvae at 200 min postinfection (Fig. S6B), suggesting that Δ*bcnA* mutant bacteria were more susceptible than Δ*bcnB* mutant bacteria to the larval humoral immune response, which is mainly driven by host antimicrobial peptides (28, 29), mirroring the different *in vitro* susceptibilities of Δ*bcnA* and Δ*bcnB* mutant bacteria to PmB. Similarly, infection of BcnA-treated *G. mellonella* larvae with *P. aeruginosa* PAO1 resulted in more rapid killing of the larvae than control or BcnB-treated larvae (Fig. 5C). We recovered significantly higher numbers of bacterial CFU from the hemolymph of BcnA-treated infected larvae (Fig. 5D), suggesting that exogenous BcnA gives infecting bacteria a survival advantage. Enhanced bacterial killing of BcnA-treated larvae was also observed for *Klebsiella pneumoniae*, *A. baumannii*, and *S. aureus* USA300 (Fig. S7C). Together, the *in vivo* infection results underpin a biological role for BCNs in giving infecting bacteria of different species a survival advantage.

### Liposoluble vitamins inhibit BcnA-mediated antibiotic capture.

**FIG 6:**
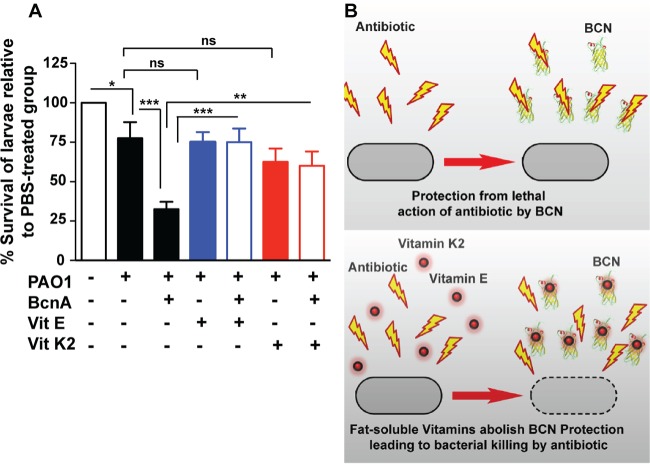
Fat-soluble vitamins inhibit BCN-mediated antibiotic binding. (A) The survival of *G. mellonella* larvae at 20 h postinfection in an *in vivo* assay of *P. aeruginosa* PAO1 protection by 1.5 µM BcnA in the presence or absence of 10 µM α-tocopherol (vitamin E) or menaquinone (vitamin K_2_) with 10 larvae per group. The results were obtained from four independent experiments and are shown as the mean percentage of larval survival in each experiment ± the SEM. The larval survival of all of the groups was 100% at zero time and 0% at 44 h, with the exception of the PBS-treated control group, which remained 100%. *, *P* < 0.05; **, *P* < 0.01; ***, *P* < 0.001 (determined by two-way ANOVA with an overall *P* value of <0.001 and the Bonferroni *post hoc* test). At the chosen sample size, the actual power of the assay to detect statistically significant effects at a significance level (alpha) of 0.05, two tailed, is 95%. ns, not significant. (B) Model of the mechanism of resistance by BcnA (top) and its inhibition by the fat-soluble vitamins (bottom).

Conceivably, molecules with superior binding affinity for BCNs than that of antibiotics should prevent BCN-mediated resistance. Since lipophilic moieties are predicted to bind deep within the BCN pocket, we tested normal dietary hydrophobic supplements recommended for several patient groups, including cystic fibrosis patients, such as the fat-soluble vitamins α-tocopherol (vitamin E) and menaquinone (vitamin K_2_). Docking of α-tocopherol showed its alkyl chain buried in the BcnA tunnel and its cyclic head placed toward the entrance, similar to the Nile red binding pose (Fig. 4E). Of note, α-tocopherol, followed by menaquinone, exhibited very low *K*_*i*_ values in Nile red displacement assays (~2 to 4 orders of magnitude lower than the *K*_*i*_ values of antibiotics), indicative of their high affinity for BcnA (Fig. 3B). This prompted us to test the BCN-inhibitory activity of fat-soluble vitamins *in vivo*. In BcnA-treated *G. mellonella* larvae infected with *P. aeruginosa* PAO1, 10 µM α-tocopherol or menaquinone significantly reduced the survival advantage of *P. aeruginosa* (Fig. 6A). This supports the notion that the protective function of BCN in infecting bacteria can be inhibited *in vivo* (Fig. 6B).

## DISCUSSION

Lipocalins are an ancient family of small proteins found in all kingdoms of life that have the ability to bind hydrophobic ligands but have different functions, depending on the cell types and organisms (30, 31). In many cases, and particularly in bacteria (18), their function is unclear despite that BCN orthologs can be found in the majority of completed genomes by data mining. In this study, we discovered that secreted BCNs contribute to antibiotic resistance by capturing and neutralizing antibiotics in the bacterial milieu. BCNs bind a range of antibiotics with diverse chemical structures, increasing antibiotic resistance *in vitro* and enhancing bacterial survival *in vivo*. The effect of BCNs on multiple antibiotics and their wide conservation in most bacterial species distinguish this mechanism from the recently discovered effect of membrane-released phospholipids on daptomycin (14), which is restricted to this particular antibiotic and to *S. aureus*.

Our structural work suggests that BCNs have two binding modes. Hydrophobic molecules like Nile red and vitamins E and K_2_ can bind in the interior of the lipocalin tunnel, while antibiotic molecules interact with the rim and their binding properties are much weaker. This suggests that antibiotic binding and scavenging are not primary functions of secreted BCNs, but these proteins may also have other, as-yet-undiscovered, roles. We propose that the antibiotic binding ability of BCNs becomes particularly relevant under conditions in which antibiotics cannot effectively kill bacteria and their presence at sublethal concentrations elicits protective bacterial stress responses. Exposure to antibiotics triggers complex and multifactorial bacterial processes involving changes in regulation, metabolism, and energy generation (32–36).

There is also a body of evidence indicating that antibiotics at sublethal concentrations can stimulate the production of reactive oxygen intermediates (37–41) and also that oxidative stress associated with pathological inflammation reduces the efficacy of antibiotics (34). The increased *bcnA* transcription upon antibiotic treatment indicates that this gene responds to antibiotic-induced stress. Recent work with the model plant *Arabidopsis thaliana* shows that two lipocalins that are related to bacterial orthologs have distinct but overlapping functions essential for protection from lipid peroxidation (42). Further, the mammalian odorant-binding protein, a soluble lipocalin, protected the bacterial cells from hydrogen peroxide-induced stress when overexpressed in *E. coli* (43), whereas *P. aeruginosa* PAO1 BcnA1(Pa) was overexpressed in response to hydrogen peroxide and paraquat (44). Donnarumma et al. (19) have recently suggested that the *Neisseria* BCN (GNA1030) is a ubiquinone-8 binding protein. Since ubiquinone-8 is a cofactor that is mainly involved in the electron transport chain (45) and has antioxidant properties (46), these authors propose a role for this protein in antioxidant defense, perhaps by delivering ubiquinone-8 to the bacterial membrane or the periplasmic space. Ubiquinones are prenylated benzoquinones. Interestingly, the majority of known crystal structures of bacterial lipocalins, including BcnA and BcnB, have been solved with octaprenyl-like molecules bound to the lipocalin tunnel, although it is not certain if the presence of this molecule corresponds to a physiological substrate or is an artifact associated with protein purification prior to crystallization. Therefore, while it may be reasonable to propose that BCNs could play a role in oxidative stress responses, the mechanisms involved remain to be elucidated.

In summary, we have uncovered a new mechanism of general antibiotic resistance operating extracellularly based on BCN-mediated antibiotic capture that provides bacteria with a selective advantage to overcome antibiotic toxicity, particularly in chronic infections, where antibiotic treatment often fails. Further, we reveal a strategy to disrupt antibiotic capture and propose liposoluble vitamins as clinically usable BCN inhibitors.

## MATERIALS AND METHODS

### Strains and reagents.

Table S2 lists the bacteria and plasmids used in this study. Bacteria were grown in LB broth, supplemented with 0.4% rhamnose when required at 37°C. *E. coli* cultures were supplemented as required with the antibiotics (final concentrations) tetracycline (30 µg/ml), kanamycin (40 µg/ml), and trimethoprim (50 µg/ml). *B. cenocepacia* cultures were supplemented as required with trimethoprim (100 µg/ml) and tetracycline (100 µg/ml). Antibiotics (Sigma) were diluted in water, except for PmB, which was diluted in 0.2% bovine serum albumin–0.01% glacial acetic acid buffer. Rifampin was dissolved in dimethyl sulfoxide (DMSO).

10.1128/mBio.00225-17.10 TABLE S2  Strains and plasmids used in this study. Download TABLE S2, PDF file, 0.1 MB. Copyright © 2017 El-Halfawy et al. 2017 El-Halfawy et al. This content is distributed under the terms of the Creative Commons Attribution 4.0 International license.

### General molecular techniques.

DNA manipulations were performed as previously described (47). T4 DNA ligase (Roche Diagnostics), Antarctic phosphatase (New England Biolabs), and restriction endonucleases were used as recommended by the manufacturers. Transformation of *E. coli* GT115 and DH5α was performed by the calcium chloride method (48). Mobilization of plasmids into *B. cenocepacia* was conducted by triparental mating (49) with *E. coli* DH5α carrying the helper plasmid pRK2013 (50). DNA amplification by PCR was performed with a C1000 thermal cycler (Bio-Rad Laboratories Ltd., Mississauga, Ontario, Canada) using *Taq* or HotStar HiFidelity DNA polymerase (Qiagen) and optimized for each primer pair. DNA sequencing was carried out at Eurofins, Huntsville, AL. The DNA sequences were analyzed with the BLAST computer program and compared to the sequenced genome of *B. cenocepacia* strain J2315. The sequence of *S. aureus* gene SAUSA300_2620 was optimized for *B. cenocepacia* codon usage and custom synthesized at Eurofins. Cloning, expression, and purification of bacteriocalins were performed as previously described (17). Transcriptional fusions to *luxCDABE* and the subsequent luminescence expression assays were performed as previously described (37). For site-directed mutagenesis, pOE16 was amplified with *Pfu* polymerase and the appropriate primer pairs; the PCR products were digested overnight with 1 U of DpnI at 37°C and then introduced into *E. coli* DH5α competent cells by transformation. Transformants were selected on LB agar plates supplemented with kanamycin; amino acid replacements were confirmed by DNA sequencing.

### Protein analysis and Western blotting.

Overnight cultures were diluted to an optical density at 600 nm (OD_600_) of 0.03 in 30 ml of fresh LB medium with or without PmB and incubated for 3.5 h at 37°C at 200 rpm. Following incubation, cells equivalent to an OD_600_ of ~0.2 were pelleted, resuspended in 30 µl of SDS-PAGE protein loading dye, and boiled to obtain whole-cell lysates. Secreted proteins were precipitated from the supernatant of the rest of the cultures with 10% trichloroacetic acid as previously described (51). Precipitated proteins were resuspended in Tris buffer at 1 M and pH 7.5. The volume of protein samples loaded onto the 16% SDS-polyacrylamide gel was normalized to the OD_600_ value. After SDS-PAGE, proteins were transferred onto nitrocellulose membranes and the membranes were blocked overnight at 4°C with Western blocking reagent (Roche Diagnostics, Laval, QC, Canada) in TBST (50 mM Tris-HCl [pH 7.5], 150 mM NaCl, 0.1% Tween 20). The primary antibody, anti-FLAG M2 monoclonal antibody (Sigma) or anti-α-subunit RNA polymerase (*E. coli*) (Neoclone, Madison, WI), was diluted to 1:15,000 in TBST and applied for 1.5 h. The secondary antibody, goat anti-mouse Alexa Fluor 680 IgG antibody (Invitrogen), was diluted to 1:15,000 and applied for 1 h. Western blot assays were developed with the LI-COR Odyssey infrared imaging system (LI-COR Biosciences, Lincoln, NE).

### Antibiotic susceptibility testing.

The inoculum of *B. cenocepacia* K56-2, the appropriate mutants, and other bacterial species was prepared by the direct colony suspension method according to CLSI (52). Cultures with an OD_600_ of 0.0008 in fresh cation-adjusted Mueller-Hinton broth (MHB) with or without the antibiotic were incubated at 37°C with medium shaking continuously in a Bioscreen C automated growth curve analyzer (MTX Lab Systems, Vienna, VA). Bacterial growth was assessed turbidimetrically at 600 nm. E-test strips (AB BioMérieux, Solna, Sweden) were applied to agar plates (17 ml of agar in an 85-mm petri dish) inoculated with test bacteria by swabbing overnight cultures diluted to an OD_600_ of 0.04; plates were then incubated at 37°C for 24 h. Alternatively, population profiling analysis was performed turbidimetrically or by CFU counting as previously described (17). For *in vitro* protection assays, *B. cenocepacia* bacteriocalins were added to LB broth at a final concentration of 1.5 µM.

### *In vitro* binding assays.

In vitro binding assays were performed as previously described (23), with a few modifications. Purified BCNs were prepared in phosphate-buffered saline (PBS, pH 7.4). Phospholipids and Nile red were prepared in DMSO. The binding of each fluorescent probe to BCNs was measured by titrating 100 µl of BCNs (1.5 µM) in a flat-bottom 96-well microtiter plate (LUMITRAC 200 White; Greiner Bio-One, Monroe, NC) with aliquots of increasing concentrations of probe until the fluorescence intensity reached a plateau, indicating that all of the binding sites were occupied. All spectra were corrected for background fluorescence determined from probe-into-buffer titrations. Fluorescence was measured with a Cary Eclipse fluorescence spectrophotometer (Varian) set at an excitation wavelength (λ_ex_) specific for each probe, as follows: Nile red, 550 nm; BODIPY phospholipids, 500 nm for fatty acyl BODIPY-labeled phosphocholine and 505 nm for head group BODIPY-labeled phosphoethanolamine. The emission spectrum for each probe was collected across the following wavelengths (λ_em_): Nile red, 590 to 750 nm; BODIPY phospholipids, 510 to 665 nm. The background-corrected binding fluorescence with each probe was fitted to a one-site binding model as previously described for human AGP (23). The equilibrium binding affinity constant for the probe-BCN complex (*K*_*d*_), the probe concentration needed to achieve half-maximum binding at equilibrium, was determined by nonlinear least-squares regression analysis of the binding isotherms with GraphPad Prism v 5.0 software (GraphPad Software, Inc.).

For probe displacement experiments, antibiotic solutions and fat-soluble vitamins (prepared in DMSO) diluted in PBS, pH 7.4, were titrated against a BCN-probe complex at a saturating concentration necessary to obtain the maximum fluorescence when bound. Probe displacement was measured as the corresponding decrease in fluorescence upon the progressive increase in the antibiotic concentration. The binding inhibition constants (*K*_*i*_s) of the test compounds were determined by nonlinear regression analysis with competition-binding equations for one-site binding calculated by GraphPad Prism v 5.0 software. The lower the *K*_*i*_ values, the higher the affinity of the molecule for BcnA. All fluorometric assays were conducted in duplicate three independent times.

### *G. mellonella* larva *in vivo* infection.

Larval infection assays were performed as previously described (53), with modifications. Overnight cultures were diluted in PBS, pH 7.4, with or without *B. cenocepacia* BCNs at a 1.5 µM final concentration to the following OD_600_s: *B. cenocepacia* and *P. aeruginosa* PAO1, 0.00004; *K. pneumoniae* Kpn18, 0.04; *A. baumannii* AB1, 0.4; *S. aureus* USA300, 0.004. The larvae were injected with 10 µl of the bacterial suspensions or sterile PBS (10 larvae/group in each experiment) with 10-µl MICROLITER syringes (Hamilton). The larvae were incubated at 30°C, and their viability was checked at regular time intervals. In similar assays, five larvae/group were sacrificed at 200 min postinfection and the hemolymph was extracted as previously described (53). The hemolymph was immediately serially diluted in PBS and plated on LB agar supplemented with 0.3% cetrimide or 200 µg/ml ampicillin–25 µg/ml PmB to quantify the CFU of *P. aeruginosa* PAO1 or *B. cenocepacia*, respectively, recovered from the infected larvae.

### Intraperitoneal infection of mice.

A clinical isolate of *P. aeruginosa* (strain Q502) was grown overnight in nutrient broth at 37°C with constant agitation. The bacteria were centrifuged at 2,000 × *g* and washed three times in sterile, endotoxin-free PBS. The bacteria were resuspended in sterile, injection-grade saline, and the inoculum was adjusted to an OD of 0.5 (*A*_550_). Female adult (8- to 12-week-old) C57BL6 mice were infected i.p. with 100 μl of the bacterial suspension. Subsequent growth of the inoculum on nutrient agar demonstrated that each animal received 10^6^ CFU. Six mice per treatment were used. This was determined by GraphPad StatMate 2.0 to ensure 80% power to detect statistically significant effects between antibiotic-treated and untreated animals at a significance level (alpha) of 0.05, two tailed. The actual power was >99%. Mice were selected at random from open stock cages (10 per cage), earmarked to allow individual identification, and then sequentially placed into treatment groups. During the course of the experiment, mice were housed in individually ventilated cages. This method of assigning animals to groups ensures an approximately equal distribution of mice from different stock cages in each group to minimize the influence of cage-to-cage variability. At the time of inoculation, the mice were treated with the standard pediatric PmB dose of 20,000 U/kg (*n* = 6), PmB and 100 µl of 25 µM BCN (*n* = 6), BCN only (*n* = 6), or a saline control (*n* = 6) by i.p. injection. The individual components injected into mice were added to the same syringe immediately before i.p. injection. Animals were culled by cervical dislocation 4 h postinoculation. That time point was selected because by that time point and under the infection conditions used, the untreated mice reach the humane endpoint and need to be culled, as defined within the UK Home Office license under which the experiments were carried out (PLL 2700). Because of the virulence of the clinical isolate and the dose of the bacterial inoculum and because the bacteria are delivered i.p., the mice rapidly succumb to the infection. In contrast, those given effective antibiotic treatment rapidly clear the infection and remain perfectly healthy. The vast divergence between the responses seen in this model provides us with the statistical power to robustly assess the microbial response to antibiotic therapies without requiring the use of a very large number of mice per group. We are therefore adhering to the reduction principle of the three R’s. The peritoneal cavity was lavaged with 3.5 ml of ice-cold, sterile, endotoxin-free PBS, and the volume recovered was recorded. Serial dilutions of the lavage fluid were plated onto cetrimide agar; and bacterial colonies were counted after 24 h of growth at 37°C. Harvesting of the samples and quantification of the bacterial burdens in the mice were done while blinded to the treatment groups. Data were not normally distributed, and there was no equal variance between groups; therefore, a nonparametric Kruskal-Wallis test was used. The mouse infection experiments carried out were assessed by the Queen’s University Belfast animal welfare and ethical review body (AWERB) committee and conducted under a license issued by the UK Home Office under the Animals (Scientific Procedures) Act 1986, amended in 2012.

### Antibiotic bioassay.

Antibiotic test solutions with or without 1.5 µM BcnA were incubated for 30 min at 37°C with rotation. The solutions were filtered through filter units with a 10-kDa cutoff by centrifugation at 7,500 × *g*, at 4°C for 10 min. The antibiotic concentrations in the filtrates were determined by spotting 5 µl onto sterile filter discs placed on agar plates swabbed with the test bacteria. Petri dishes (15-cm diameter) containing 40 ml of LB agar were swabbed with bacterial suspensions with an OD_600_ of 0.04. The plates were incubated at 37°C for 24 h. Each plate included four discs containing standard concentrations of the antibiotic alongside the discs impregnated with test and control antibiotic solutions. *E. coli* DH5α was used for bioassays of PmB, norfloxacin, ceftazidime, and gentamicin, and *S. aureus* USA300 was used for bioassays of rifampin. The theoretical disc contents of the test antibiotic solutions were 10, 5, 2, 30, and 10 µg for PmB, rifampin, norfloxacin, ceftazidime, and gentamicin, respectively. Standard antibiotic discs contained a 2-fold higher concentration than, the same concentration as, or a 2- or 4-fold lower concentration than the test antibiotic discs. After incubation, the clear zones of inhibition were measured and the antibiotic concentrations were determined from standard curves constructed from the standard antibiotic discs.

### Structure determination.

BcnA and BcnB were purified by fast protein liquid chromatography and concentrated to ~20 mg/ml in a mixture of 20 mM Tris (pH 7.5) and 100 mM NaCl (1% DMSO for BcnB) with >95% purity. Protein solutions were mixed 1:1 with mother liquor, and crystals were grown at room temperature. BcnA crystals grew with 0.1 M Tris (pH 8.5) and 2.4 M ammonium sulfate (final pH of 8.0) as the mother liquor. BcnB crystals grew with 0.1 M HEPES (pH 6.5)–26% polyethylene glycol 6000 as the mother liquor. Data sets were collected on beamline 08ID-1 at Canadian Light Sources (54), integrated with iMOSFLM (55), and scaled with AIMLESS (56) from CCP4. Phases for BcnA and BcnB were obtained with Phaser.MRage (57) from PHENIX with PDB accession no. 2FGS and 1WUB, respectively, as the search models. Both structures were initially built with AutoBuild (58) from PHENIX and then manually built with Coot (59). The BcnA and BcnB structures were refined with phenix.Refine (60) from PHENIX and REFMAC5 (61) from CCP4, respectively, with translation-libration-screw motion refinement for both. Figures were generated with Pymol, version 1.8 (https://www.pymol.org/). Data collection and refinement statistics are provided in Table S1 in the supplemental material. SEC-MALS experiments were conducted to assess the oligomeric solution state of BcnA and BcnB as previously described (62) with proteins diluted to 1 mg/ml in 20 mM Tris (pH 7.5)–100 mM NaCl.

### Computational methods.

All of the following codes can be obtained under license agreement: AMBER 12 and AmberTools (http://www.ambermd.org), Maestro Suite (Schrödinger, Cambridge, MA), AutoDock 4.2.2, and AutoDockTools (http://autodock.scripps.edu; free software). Amber and Maestro have Academic fees. We studied the stability of the two X-ray structures of BcnA and BcnB, as well as the D82A-D93A mutant, by MD simulations as implemented in AMBER 12. The initial model of D82A-D93A was built with AmberTools. Missing hydrogen atoms were added, and the protonation state of ionizable groups was computed by using Maestro Protein Preparation Wizard, version 9.3 (Schrödinger, Cambridge, MA). Atom types and charges were assigned according to AMBER ff10 force field (63). The three molecular systems were hydrated by using cubic boxes containing explicit TIP3P water molecules extending 10 Å away from any protein atom for simulating the aqueous environment with the help of AmberTools with added counterions to neutralize the system. Before the MD simulations, the two systems were equilibrated under the following protocol: an initial 8,000 steps of steepest-descent minimization, followed by heating of the system with position restraint (force constant of 20 kcal mol^−1^ Å^−2^) for all protein atoms during 10 ps of MD simulation increasing the temperature from 100 K to 300 K plus an additional 15 ps at a constant temperature of 300 K. Position restraint was gradually decreased for 100 ps at a constant 300 K, until the full system was under no restraint with constant temperature (300 K) and pressure (1 atm). After equilibration, 20 ns of MD simulation was run at a constant temperature (300 K) and pressure (1 atm). Short- and long-range forces were calculated every one and two time steps, respectively (each time step was 2.0 fs), constraining the covalent bonds involving hydrogen atoms to their equilibrium values. Long-range electrostatic interactions were accounted for by means of the particle mesh Ewald approach applying periodic boundary conditions. The RMSD as a function of time with respect to the starting structure of the α-C atoms was computed with CPPTRAJ (64). The three-dimensional (3D) coordinates of the structures of BcnA and BcnB subjected to the equilibration protocol described above were used for docking purposes. The two models were prepared for docking calculations by adding Kollman charges (65) with the help of AutoDockTools.

3D coordinates of norfloxacin, PmB, ceftazidime, gentamicin, Nile red, and α-tocopherol were built in Corina (66) from the SMILES code. The 3D structure of rifampin was extracted from the crystallographic structure PDB accession no. 1LSV. The structures of the seven ligands were protonated at pH 7.0 with Epik (67) and then optimized with MMFFs force field by with MacroModel version 9.9 (Schrödinger, Cambridge, MA). Additionally, the structure of PmB was subjected to 100 ps of MD simulation at 300 K. Ligands were prepared for docking calculations with AutoDockTools by adding Gasteiger charges (68) and setting all rotatable bonds free to move during the docking calculation.

Docking calculations of all compounds were performed by means of AutoDock 4.2.2 (69). Analysis was performed with the help of AutoDockTools. The grid point spacing was set at 0.375 angstroms, and a hexahedral box was built with *x*, *y*, and *z* dimensions of 21.00, 26.25, and 27.75 angstroms centered in the binding site of the protein. Two hundred runs with the Lamarckian genetic algorithm were performed with a population size of 100 and 250,000 energy evaluations. Side chains of residues Y85, W94, and Q41 were considered flexible during the docking protocol.

### BCN consensus motif determination.

A subset of 187 curated BcnA homologues from different bacterial species and families were used to obtain a consensus motif generated by the Gapped Local Alignment Motifs GLAM2 tool (70). This motif was verified by analyzing an alignment of 1,995 BCN homologues with CLUSTAL-omega and visualized with JalView. In addition, upon submitting the predicted motif to the GLAM2Scan database (70) against *B. cenocepacia*, *P. aeruginosa* PAO1, *M. tuberculosis* H37Rv, and *S. aureus* USA300, the correct homologues only were detected as BCNs for each of the organisms.

### Statistical analyses.

Statistical analyses were conducted with GraphPad Prism 5.0. All of the results shown are the mean ± the standard error of the mean (SEM) unless otherwise stated. Unless otherwise stated, data were assumed to follow a Gaussian distribution as determined by the D’Agostino-Pearson omnibus K2 normality test whenever possible, and hence, *t* tests and analysis of variance (ANOVA) were used. An unpaired *t* test was used to compare the means of two unmatched groups. A paired *t* test was used to compare the means of two matched groups, assuming that the before-and-after differences follow a Gaussian distribution. The sample size was chosen with GraphPad StatMate 2.0 to ensure a minimum of 80% power to detect statistically significant effects at a significance level (alpha) of 0.05, two tailed. However, the actual power of most of the assays was ≥90% and in many cases exceeded 99%. In the case of MIC assays, the experiments were repeated three independent times and the experiment showing the lowest fold change (if applicable) was reported.
