# Supplementary figures and images for: Antibiotic Capture by Bacterial Lipocalins Uncovers an Extracellular Mechanism of Intrinsic Antibiotic Resistance
Source: mBio. 2017 Mar 14;8(2):e00225-17. doi: 10.1128/mBio.00225-17 (PMC5350466; doi:10.1128/mBio.00225-17)

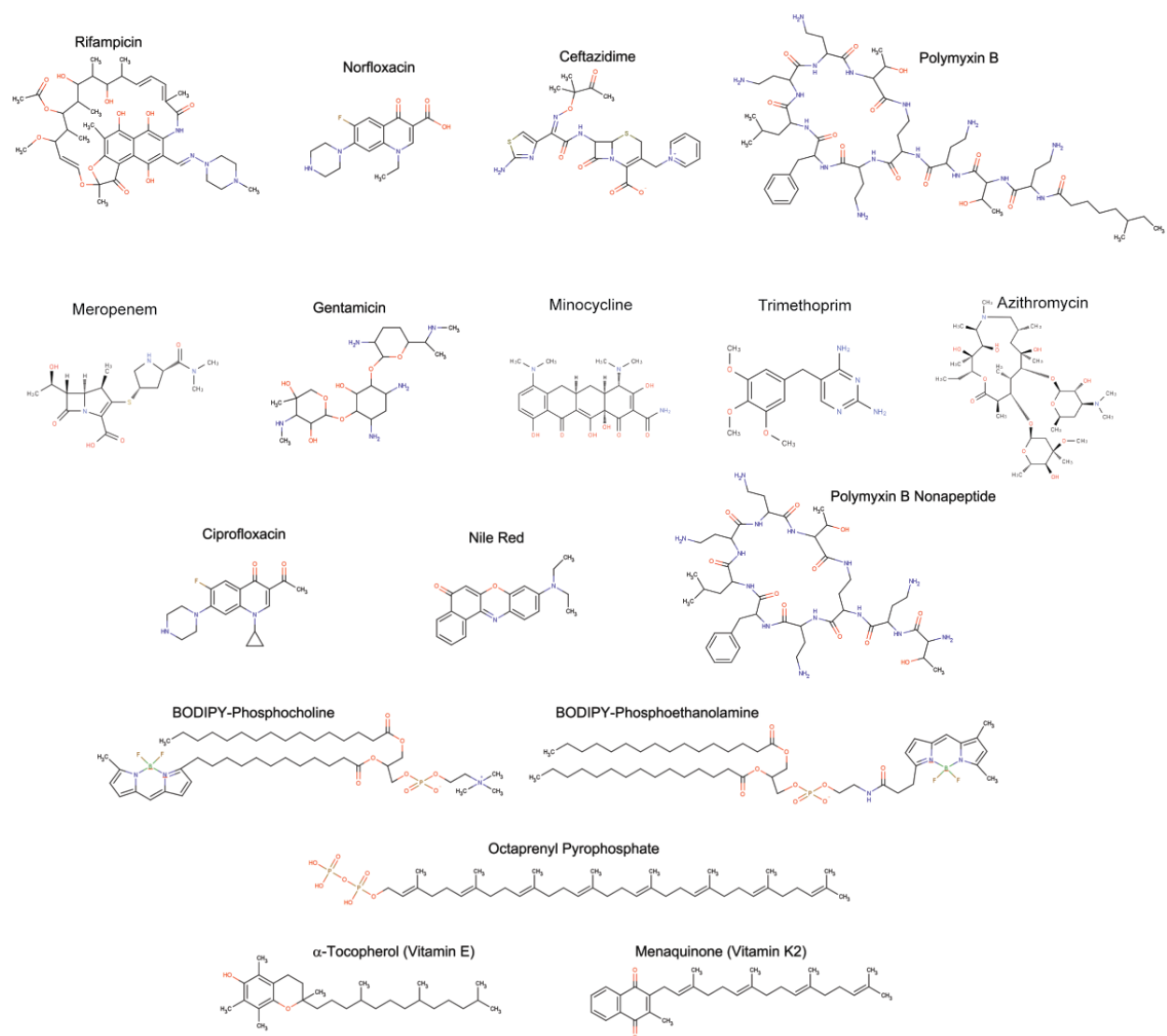

**Fig. S1.** Chemical structures of antibiotics and chemicals used in this study.

Supplement: FIG S1 [file mbo002173230sf1.pdf]
