# Supplementary material for: Antibiotic Capture by Bacterial Lipocalins Uncovers an Extracellular Mechanism of Intrinsic Antibiotic Resistance
Source: mBio. 2017 Mar 14;8(2):e00225-17. doi: 10.1128/mBio.00225-17 (PMC5350466; doi:10.1128/mBio.00225-17)
Supplement: FIG S2 [file mbo002173230sf2.pdf]

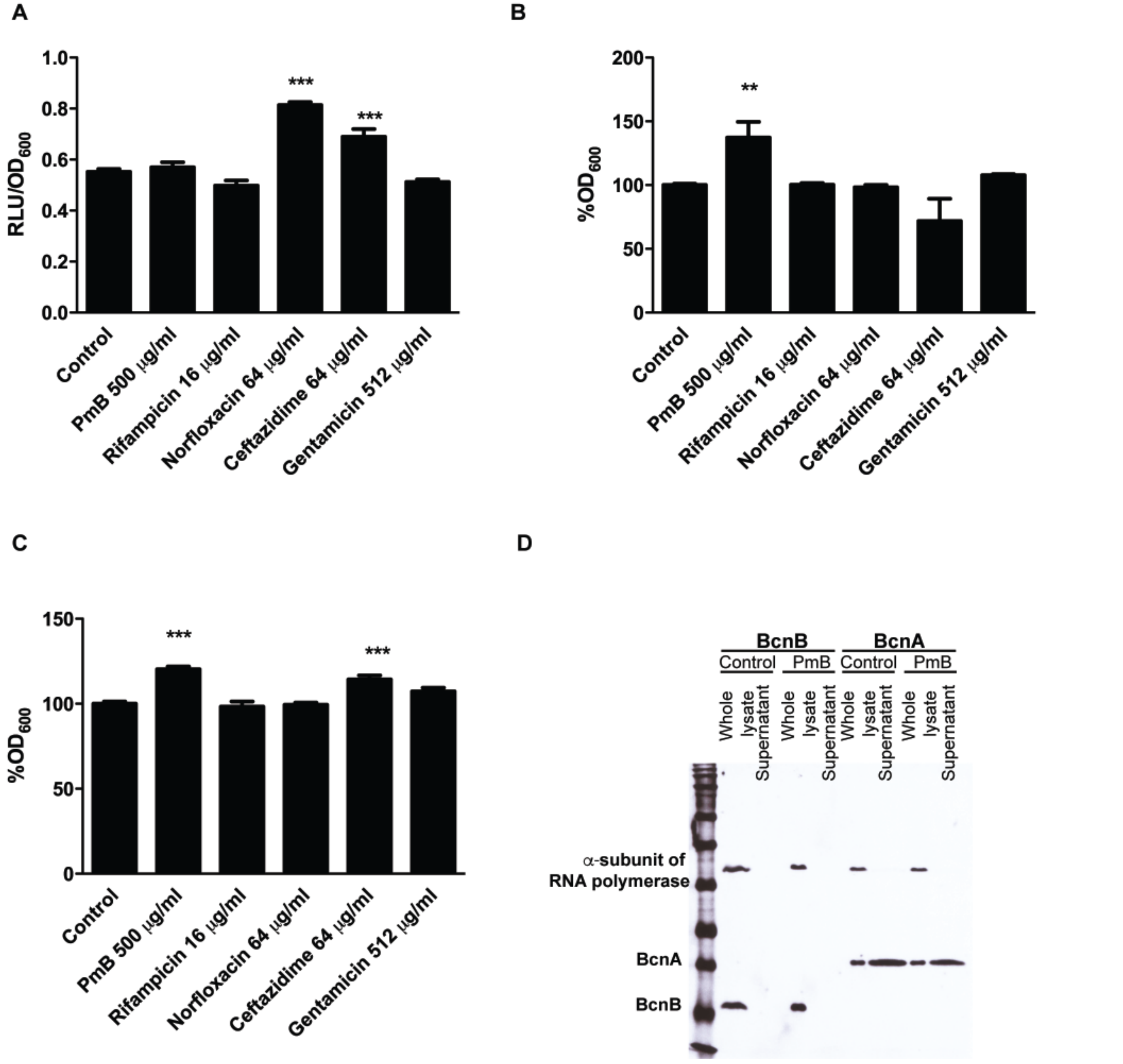

Fig. S2. Expression and secretion profiles of BcnA and BcnB. (A) Luciferase expression assay of BcnB and the relative growth of cells in the luminescence expression assay of the different *B. cenocepacia* BCNs in response to antibiotics at 3 h. Expression of BcnB (OME60). n=6 from 2 different clones. Results are shown as mean of relative light units RLU/OD_{600} ±SEM. (B) Growth in the expression assay of BcnB (OME60). n=6 from 2 different clones. The mean OD_{600} of the control is 0.0963. (C) Growth in the expression assay of BcnA and the associated CybB (OME61) shown in Fig. 1B. n= 6 from 2 different clones. Results are shown as mean of percentage of OD_{600} relative to the control (untreated K56-2 background) ±SEM. The mean OD_{600} of the control is 0.0998. * $p<0.05$, ** $p<0.01$ and *** $p<0.001$ from one-way ANOVA tests of overall $p<0.001$ and Bonferroni's post-hoc test compared to the respective untreated control conditions. (D) BcnA is the only secreted *B. cenocepacia* BCN; proteins (carrying a C-terminal FLAG-tag) were detected in whole cell lysates and supernatants of control untreated cultures or cultures treated with 2 μg/ml PmB by Western blot using anti-FLAG antibody. The α-subunit of the RNA polymerase was used as a control for cell lysis. Representative of 3 independent experiments.
