# Supplementary material for: Antibiotic Capture by Bacterial Lipocalins Uncovers an Extracellular Mechanism of Intrinsic Antibiotic Resistance
Source: mBio. 2017 Mar 14;8(2):e00225-17. doi: 10.1128/mBio.00225-17 (PMC5350466; doi:10.1128/mBio.00225-17)
Supplement: FIG S3 [file mbo002173230sf3.pdf]

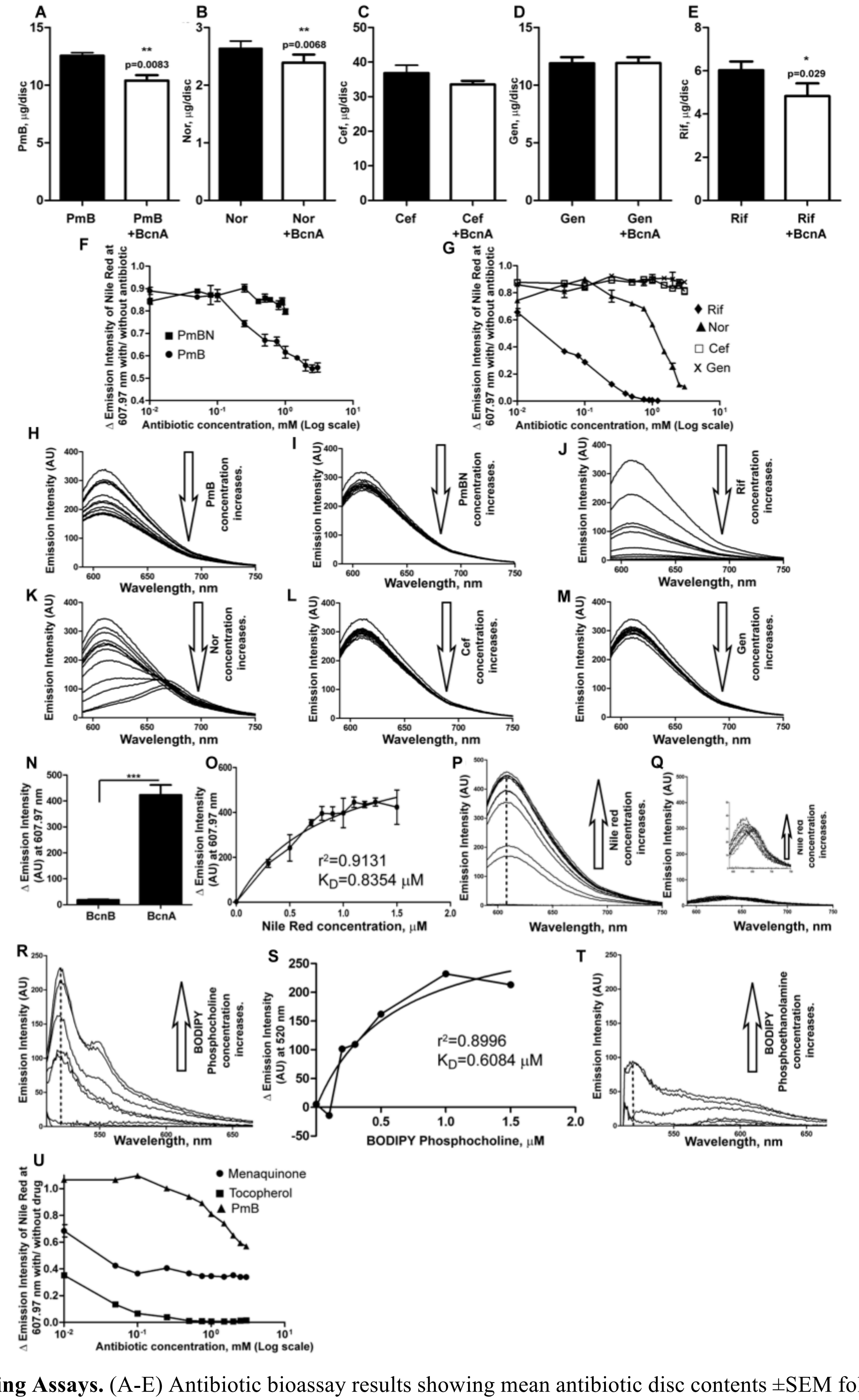

Fig. S3. Binding Assays. (A-E) Antibiotic bioassay results showing mean antibiotic disc contents ±SEM following mixing with BcnA and passing through centrifugal filter units of MWCO 10 KDa compared to control antibiotic solutions. * p<0.05,

and ** $p<0.01$ from paired student's t-tests compared to the respective control conditions. n=3 from 3 independent experiments. At this sample size, the actual power of the assay to detect statistically significant effects at significance level (alpha) of 0.05, two-tailed is >99%. (F-G) Binding displacement assays of Nile Red from 1.5 µM BcnA-1.5 µM Nile Red complex by antibiotics and other chemicals. 3 independent experiments, n=5. (H-M) Binding displacement isotherms showing the displacement of Nile Red by different antibiotics from its complex with BcnA. Mean of n=3 from a representative of 3 independent experiments. (N) The affinity of 1.5 µM BcnA compared to that of 1.5 µM BcnB to bind 1.5 µM Nile Red, n=5 from 3 independent experiments. *** $p<0.001$ from unpaired two-sided Student's t-test. (O) *In vitro* binding assay of 1.5 µM BcnA to Nile Red in PBS, 3 independent experiments, n=5. (P and Q) Binding isotherms from the fluorometric assays showing the interaction between BcnA (P) or BcnB (Q) and Nile Red. Mean of n=3 from a representative of 3 independent experiments. (R-T) Nile red binding affinity of BcnA to BODIPY phosphocholine (R and S) and BODIPY phosphoethanolamine (T), n=4 from 2 independent experiments. (U) Binding displacement assays of Nile Red from 1.5 µM BcnA-1.5 µM Nile Red complex by vitamins. n=5 from 3 independent experiments
