# Supplementary material for: Antibiotic Capture by Bacterial Lipocalins Uncovers an Extracellular Mechanism of Intrinsic Antibiotic Resistance
Source: mBio. 2017 Mar 14;8(2):e00225-17. doi: 10.1128/mBio.00225-17 (PMC5350466; doi:10.1128/mBio.00225-17)
Supplement: TEXT S1 [file mbo002173230s1.pdf]

SUPPLEMENTAL RESULTS

Antibiotic capture by bacterial lipocalins uncovers an extracellular mechanism of intrinsic antibiotic resistance

Omar M. El-Halfawy[a,b], Javier Klett[c], Rebecca J. Ingram[d], Slade A. Loutet[e], Michael E. P. Murphy[e], Sonsoles Martín-Santamaría[c], and Miguel A. Valvano[a,d,1]

X-ray crystallography and SEC-MALS. BCNs generally consist of an extended, eight-stranded, antiparallel β-barrel with a lipocalin fold, able to host lipophilic ligands inside a lipophilic tunnel, together with a characteristic α-helix (1). The crystal structure of BcnA was solved to 1.4 Å resolution in space group $P6_1$ (Table S1). The asymmetric unit contained two molecules of BcnA. Recombinant BcnA lacked the predicted signal peptide (first 26 amino acids) of the full-length protein. In both chains, the model contained N-terminal histidyl and methionyl residues encoded from the expression plasmid, residues D27 through T185 of BcnA; only the terminal amino acid (K186) was not modeled. There are few contacts between the two protein chains in the asymmetric unit. The PDBePISA server (2) predicted that the molecules in the asymmetric unit are monomers in solution. The oligomeric solution state of BcnA was assessed using SEC-MALS. Recombinant BcnA, after cleavage of the 6X-His tag, has a predicted molecular mass of 17.4 kDa. The measured mass of BcnA in solution was 18 kDa (Fig. S4D). Based on these results we concluded that BcnA is a monomeric protein (Fig. 4A). Within the interior cavity of BcnA, continuous density was observed with a shape similar to that observed for isoprene-based molecules identified in homologous BCN structures (PDB IDs 1Y0G, 3Q34, 2X32, 2X34, 1WUB). We have modeled this density as octaprenyl pyrophosphate (OTP) (Fig. S4A) as observed in other BCN proteins after recombinant expression in *E. coli* (3, 4). Unambiguous density for the isoprene chain of OTP is observed in the BcnA structure; however, little density is present corresponding to the pyrophosphate portion of OTP. The isoprene tail of OTP makes numerous hydrophobic contacts with amino acid side chains located inside the BcnA barrel. The binding of these lipophilic ligands to the BCN homologues is likely due to recombinant expression in *E. coli*; hence these molecules might not be predictive of the functions of these BCN homologues.

The crystal structure of BcnB was solved to 1.6 Å resolution in space group $P2_1$ (Table S1) with four molecules of BcnB in the asymmetric unit. Recombinant BcnB lacked a predicted signal peptide (first 22 amino acids) of the full-length protein. For chains B, C, and D, residues S24 to Q192 were modeled, while for chain A residues A25 to Q192 were modeled. All chains lack the C-terminal amino acid (Q193). PDBePISA predicted a stable dimer reconstructed through crystallographic symmetry that buries ~2840 $Å^2$ of surface area, 15% of the solvent accessible surface area of each protomer. The mass of BcnB in solution was measured to be 38.1 kDa (Fig. S4E) by SEC-MALS. Recombinant BcnB, after cleavage of the 6X-His tag, has a predicted molecular mass of 19.2 kDa, indicating that BcnB is dimeric (Fig. 4B). Again, an interior of the cavity of BcnB contains continuous density that was modeled as OTP whose isoprene tail also makes numerous hydrophobic contacts with amino acid side chains located inside the BcnB barrel (Fig. S4B). In this case, the density supports both the isoprene chain and the pyrophosphate portions of OTP.

Structural alignments by the DALI server (5) indicated that both BcnA and BcnB were most similar to *E. coli* YceI (PDB ID 1Y0G). For BcnA, 156 residues were aligned (24% sequence identity) with a root mean squared deviation (rmsd) of 1.5 Å. For BcnB, 165 residues were aligned (33% sequence identity) with a

rmsd of 1.7 Å. BcnA and BcnB also shared structural similarity (Z-score > 3.0) to eukaryotic lipocalins such as porcine odorant binding protein (PDB ID 1DZM) and plasma retinol-binding protein (PDB ID 1KT6), as well as avidin (PDB ID 2A5C). These results confirm that despite low sequence identity, BcnA and BcnB are members of the BCN family and both possess a lipocalin fold. A structural alignment of BcnA and BcnB (Fig. 4C) shows that the overall folds are very similar (148 residues aligned with an rmsd value of 1.66 Å) despite only sharing 22% sequence identity. The largest structural differences in the proteins are in two of loops at the open ends of the β-barrels. In BcnB these form a longer tunnel with amino acid residues extending past the OTP pyrophosphate (Fig. S4B), while in BcnA the OTP pyrophosphate extends past the end of the protein barrel. BcnB residues H42, W49, S84 (*via* a water-bridge), and K86 provide a hydrogen bonding network for the OTP pyrophosphate (Fig. S4C), while BcnA does not provide such a network.

Extended computational studies of BcnA, BcnB, and complexes with the studied ligands. We conducted structure-function analyses of BcnA to propose a plausible binding mode for antibiotics. The stability of the BcnA and BcnB models was tested by running 20 ns of molecular dynamics (MD) simulations with AMBER 12 (http://ambermd.org/). RMSD deviations and RMS fluctuations (Fig. S9a-b) show that the two systems reach stability along the MD simulation.

Docking of antibiotics in addition to Nile Red and α-tocopherol was performed in the BcnA X-Ray structure, and binding poses were predicted. In addition, flexibility of the protein was also taken into account by allowing some residues to be flexible during the docking calculations. Docking results are shown for BcnA in Fig. 4D-E and Fig. S4F-I. Interestingly, two distinct binding modes were predicted for BcnA: antibiotics were predicted to bind at the rim of the lipocalin pocket whereas more lipophylic molecules such as Nile Red bind deeper inside the lipophylic tunnel.

All tested antibiotics shared ionic interactions with several polar residues, mainly K40, T46, D82, Q88, Y85, D93, and E165 (Fig. 4D and Fig. S4F-I). In addition to ionic interactions, other interactions were observed between the aromatic moieties present in PmB, rifampicin, norfloxacin, and ceftazidime, and lipophylic residues such as I171, Y85, M42, M84, V89, W94 and W166. Gentamicin does not possess any aromatic moieties, matching with its weak binding to BcnA. However, the inability to bind BcnA cannot be correlated exclusively to the absence of aromatic moieties; other factors such as entropic effects from solvation and/or conformational factors may be involved.

Docking calculations of Nile Red and the fat-soluble α-tocopherol led to the prediction of the ligand deeply binding inside the lipophylic tunnel, establishing lipophylic interactions. In case of Nile Red, we predicted two alternative binding modes in BcnA, one with the diethylamino group pointing towards the entrance of the pocket, and other one with the diethylamino group pointing towards the interior of the protein. In the case of α-tocopherol, it was found to be mainly docked with the alkyl chain buried into the pocket and the cyclic head placed towards the entrance of the pocket (Fig. 4E). The OH group from the chromanol head was not found to establish any preferred polar interaction over the different predicted poses.

BcnA model and docking calculations suggested D82 and D93 as important residues for the structure and function of BcnA. We found that in the wild type BcnA, the D82 carboxylate group establishes stable hydrogen bonds with the backbone NH groups of M84 and Y85 from helix D82-A89, and D93 carboxylate group establishes a stable hydrogen bond with the side chain OH group of T163. Prompted by this finding, we modeled the BcnA D82A-D93A mutant from the crystallographic structure, and submitted to MD simulations. The stability of the BcnA D82A-D93A double mutant model was tested by running 20 ns of MD simulation with AMBER 12. RMSD deviations and RMS fluctuations (Fig. S6A-B) show that the system reached stability along the MD simulation. We could only observe a slight lower fluctuation in the case of BcnA D82A-D93A mutant: a 3Å of RMS fluctuation is reached in the region of the V38-E47 loop in the case of the D82A-D93A mutant while, in the case of the WT BcnA, a 4 Å of RMS fluctuation is

observed for the same loop, in accordance with the B-factors extracted from the crystallography structures. By contrast, in the case of the MD simulation of the BcnB, the equivalent F38-R50 loop showed a 2Å of RMS fluctuation. To study the reason that causes this lower fluctuation in the BcnA mutant model, we inspected the MD simulations in detail. A higher mobility of the atoms belonging to the V38-E47 loop was found in the case of the wild type, as deduced from the RMS fluctuations of each residue (Fig. S6A-B). We observed that, in the case of the BcnA D82A-D93A mutant, the V38-E47 loop created new interactions between the backbone of M42 and S170, and Q41 and A173, maybe thus explaining this lower fluctuation.

In the D82A-D93A mutant, the absent carboxylate group of D82 is replaced by the backbone CO group from A82, allowing the hydrogen bonding to NH groups from M84 and Y85. This new situation might affect the overall BcnA structure and, very likely, the binding of antibiotics since D82 has been identified by our docking calculations as an important residue for antibiotic anchorage. Furthermore, in the case of D93 mutation, the absence of the hydrogen bond between the carboxylate group and the T163 side chain is not replaced by other equivalent interaction, allowing the approach of T163-I171 loop towards the V38-E47 loop, leading to two novel hydrogen bonds: one between the M42 NH group and the S170 CO group, and another one between the Q41 CO group and the A173 NH group (Fig. S6C-E). Together, this relatively different arrangement for the BcnA D82A-D93A mutant led to a reduced flexibility of the V38-E47 loop along the MD simulation time (Fig. S6C-E). The higher mobility of this V38-E47 loop could resemble the required movement of a similar loop in other member of the lipocalin family (the lipocalin type prostaglandin D synthase), where the conformational change of the Y107-S114 loop allows the change between the open/closed conformers (6). Nevertheless, the higher flexibility observed for the wild type structure could account for a better ability to bind ligands. Taking always into account the limitation of working with computational simulations, these observed changes might point out to a structural role for D82 and D93, in addition to their putative functional role in the binding of the antibiotics. To further investigate the role of these two residues, site-directed mutants with alanine replacements of these residues were prepared. D82 and to a less extent D93 were demonstrated to be important for the binding interaction of ligands (exemplified by Nile Red) to BcnA (Fig. S5A-B). Docking of Nile Red and α-tocopherol into the BcnA D82A-D93A mutant (by following the general docking protocol with the minimized average structure from the ns 2.5 to the ns 5 of the MD simulation) did not lead to any binding pose inside the lipophilic tunnel. This agrees with the experimental results obtained with the BcnA D82A-D93A mutant protein.

Since BCNs are highly conserved among bacteria sharing the characteristic lipocalin tertiary structure, we determined the consensus motif of this protein family (Fig. S5C) (7).The D93 residue of BcnA of *B. cenocepacia* was highly conserved. Aspartate residues at distance from D93 comparable to the distance to D82 in *B. cenocepacia* BcnA were found in the motif (7). Together, this suggests that D82 and D93 may have a structural role to maintain the 3D structure and the opening of the lipophilic tunnel of BcnA, and also they may be proposed as key residues to interact with antibiotics, thus playing an essential role in the resistance mechanism mediated through BCNs. This mode of interaction between BCN and antibiotics could be common among this large family of conserved bacterial proteins.

1. **Bishop RE.** 2000. The bacterial lipocalins. Biochim Biophys Acta **1482:**73-83.

2. **Krissinel E, Henrick K.** 2007. Inference of macromolecular assemblies from crystalline state. J Mol Biol **372:**774-797.

3. **Handa N, Terada T, Doi-Katayama Y, Hirota H, Tame JR, Park SY, Kuramitsu S, Shirouzu M, Yokoyama S.** 2005. Crystal structure of a novel polyisoprenoid-binding protein from *Thermus thermophilus* HB8. Protein Sci **14:**1004-1010.

4. **Vincent F, Molin DD, Weiner RM, Bourne Y, Henrissat B.** 2010. Structure of a polyisoprenoid binding domain from *Saccharophagus degradans* implicated in plant cell wall breakdown. FEBS Lett **584:**1577-1584.

5. **Holm L, Rosenström P.** 2010. Dali server: conservation mapping in 3D. Nucleic Acids Res **38:**W545-549.

6. **Kumasaka T, Aritake K, Ago H, Irikura D, Tsurumura T, Yamamoto M, Miyano M, Urade Y, Hayaishi O.** 2009. Structural basis of the catalytic mechanism operating in open-closed conformers of lipocalin type prostaglandin D synthase. J Biol Chem **284:**22344-22352.

7. **Frith MC, Saunders NF, Kobe B, Bailey TL.** 2008. Discovering sequence motifs with arbitrary insertions and deletions. PLoS Comput Biol **4:**e1000071.
