# Supplementary material for: Antibiotic Capture by Bacterial Lipocalins Uncovers an Extracellular Mechanism of Intrinsic Antibiotic Resistance
Source: mBio. 2017 Mar 14;8(2):e00225-17. doi: 10.1128/mBio.00225-17 (PMC5350466; doi:10.1128/mBio.00225-17)
Supplement: FIG S4 [file mbo002173230sf4.pdf]

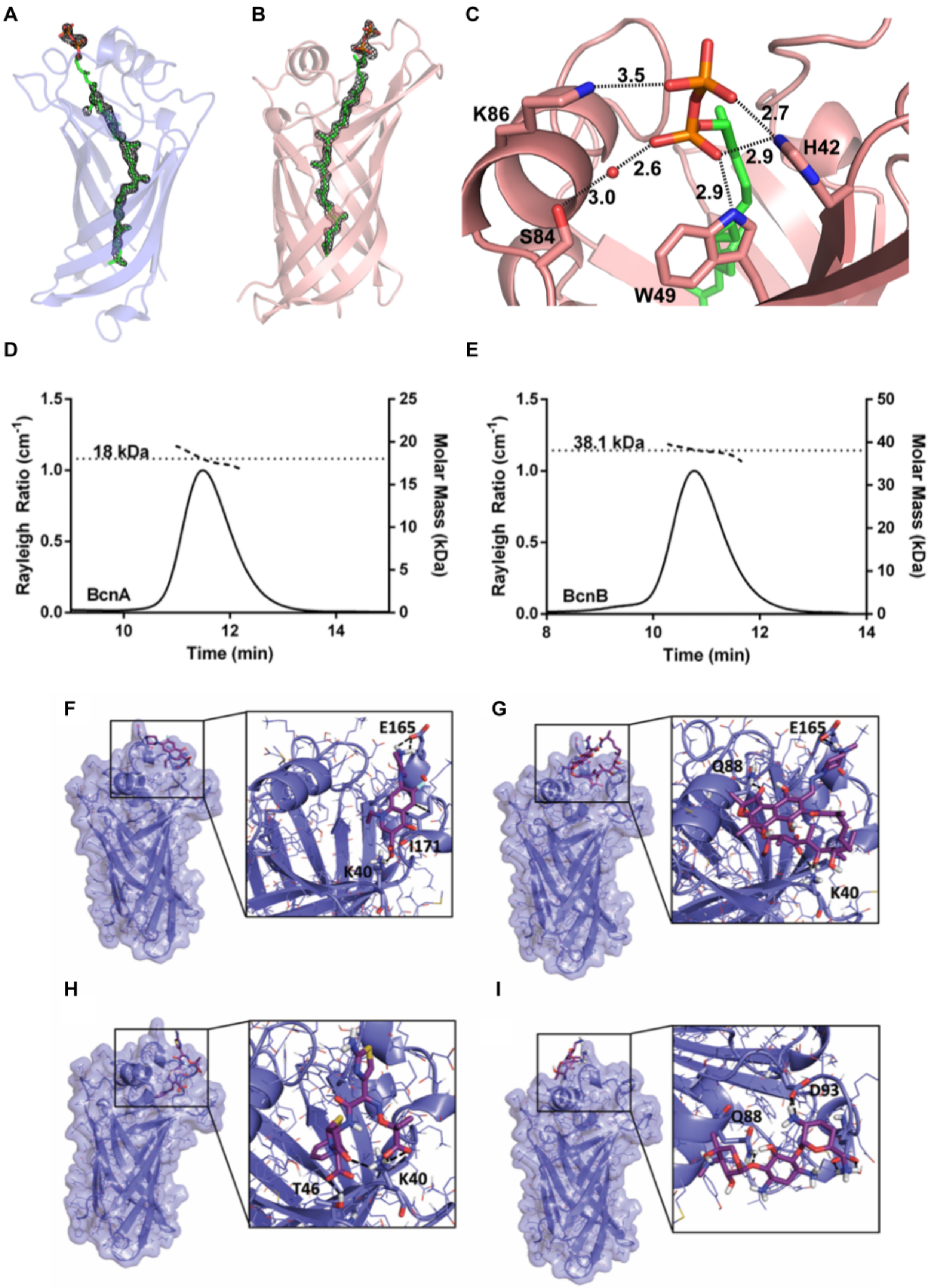

Fig. S4. BcnA and BcnB macromolecular structures and docking binding models of antibiotics into the BcnA structure. BcnA (A) and BcnB (B) possess electron density within the interior of the protein cavities that was modeled as octaprenyl pyrophosphate (OTP; carbon, green; phosphate, brown; oxygen, red). OTP omit maps (Fo-Fc) are contoured at

3.0 σ and shown in black mesh. (C) The cavity opening of BcnB provides a hydrogen-bonding network to the OTP pyrophosphate via amino acids H42, W49, K86, and S84 through a water (red sphere) bridge. These residues are shown as stick figures (carbon, peach; nitrogen, blue; oxygen, red). Hydrogen bonds are represented as dotted lines with distances between the hydrogen bond donor and acceptor atoms shown in Å. The molar mass determination of BcnA (D) and BcnB (E) in solution were determined by SEC-MALS; the elution profiles (solid lines) represent the intensity of scattered light and are expressed as rayleigh ratios. The measured molar masses (dashed line) were constrained to the single elution peak for each protein. The dotted lines represent the final calculated mass for each protein. The BcnA crystallographic structure was used to perform molecular docking experiments. Selected docked binding modes of the antibiotics norfloxacin (F), rifampicin (G), ceftazidime (H), and gentamicin (I) into the BcnA structure (displayed in blue) and main residues interacting with the different ligands (CPK colors) are shown.
