# Supplementary material for: Antibiotic Capture by Bacterial Lipocalins Uncovers an Extracellular Mechanism of Intrinsic Antibiotic Resistance
Source: mBio. 2017 Mar 14;8(2):e00225-17. doi: 10.1128/mBio.00225-17 (PMC5350466; doi:10.1128/mBio.00225-17)
Supplement: TABLE S1 [file mbo002173230st1.pdf]

Table S1. X-ray crystallography data collection and refinement statistics

Parameter	BcnA	BcnB
**Data Collection***		
Resolution Range (Å)	35.9 - 1.4 (1.43 – 1.4)	37.3 - 1.6 (1.63 – 1.6)
Space Group	$P6_1$	$P2_1$
Unit cell dimension (Å)	a = 93.97, b = 93.97, c = 76.66, γ = 120°	a = 51.2, b = 101.2, c = 63.5, β = 97.2°
Unique Reflections	75,559 (4,004)	82,019 (3,495)
Completeness (%)	99.9 (99.4)	97.3 (78.7)
CC1/2	0.999 (0.829)	0.997 (0.892)
Average $I/\sigma I$	17.1 (3.0)	11.3 (3.3)
Redundancy	10.5 (5.7)	3.8 (3.1)
R_{merge}	0.061 (0.492)	0.056 (0.265)
**Refinement**		
R_{work} (R_{free})	0.156 (0.183)	0.158 (0.192)
Number of water molecules	274	482
Overall B-factor (Å^2)	28.4	17.1
r.m.s.d.		
Bond lengths (Å)	0.01	0.025
Bond angles (°)	1.46	2.44
Ramachandran Plot (%)		
In most favorable	96.0	96.9
In disallowed	0.3	0.8

*Data collection statistics in parentheses represent the highest resolution shells. Coordinates for the X-ray crystal structures of BcnA and BcnB have been deposited to the Protein Data Bank with Accession Codes 5IXH and 5IXG, respectively.
