# Supplementary material for: Antibiotic Capture by Bacterial Lipocalins Uncovers an Extracellular Mechanism of Intrinsic Antibiotic Resistance
Source: mBio. 2017 Mar 14;8(2):e00225-17. doi: 10.1128/mBio.00225-17 (PMC5350466; doi:10.1128/mBio.00225-17)
Supplement: FIG S5 [file mbo002173230sf5.pdf]

A

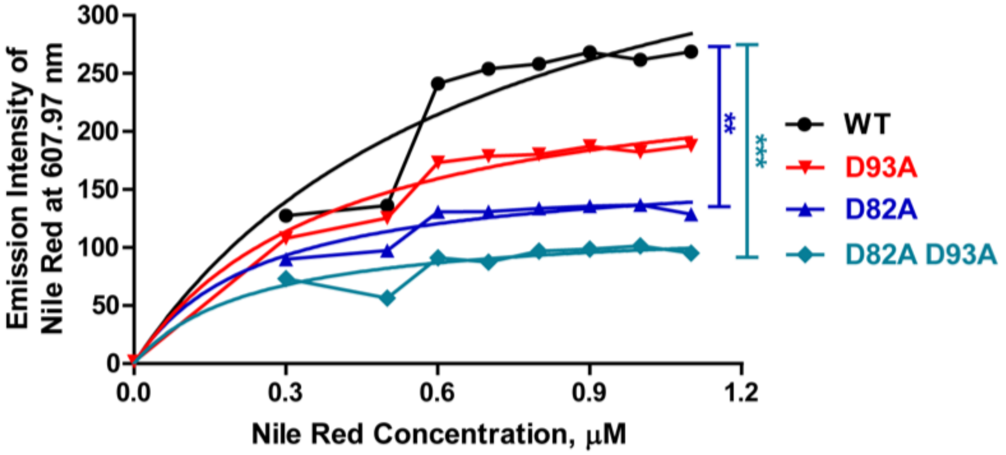

B

One site - Specific binding	WT	D93A	D82A	D82A D93A
K_D ±SE, µM	**0.6998** ±0.1786	**0.4001** ±0.1288	**0.2487** ±0.06661	**0.2354** ±0.1256
r^2	0.9291	0.8755	0.9198	0.7502

C

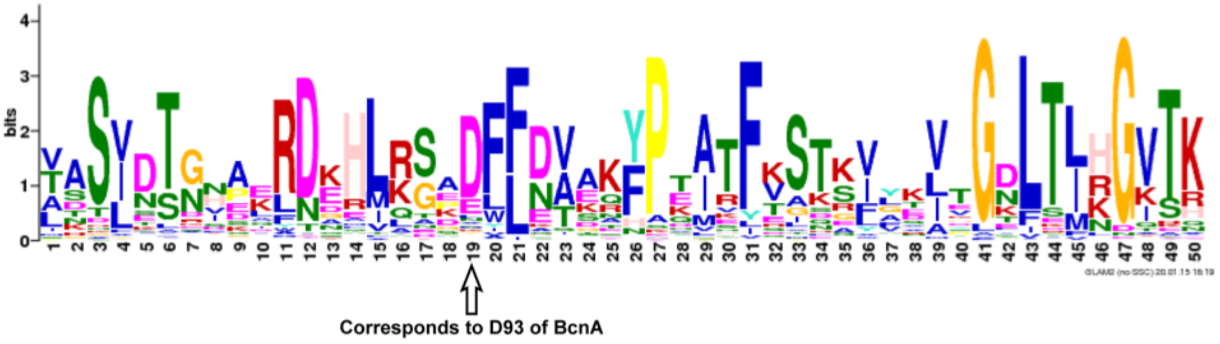

D

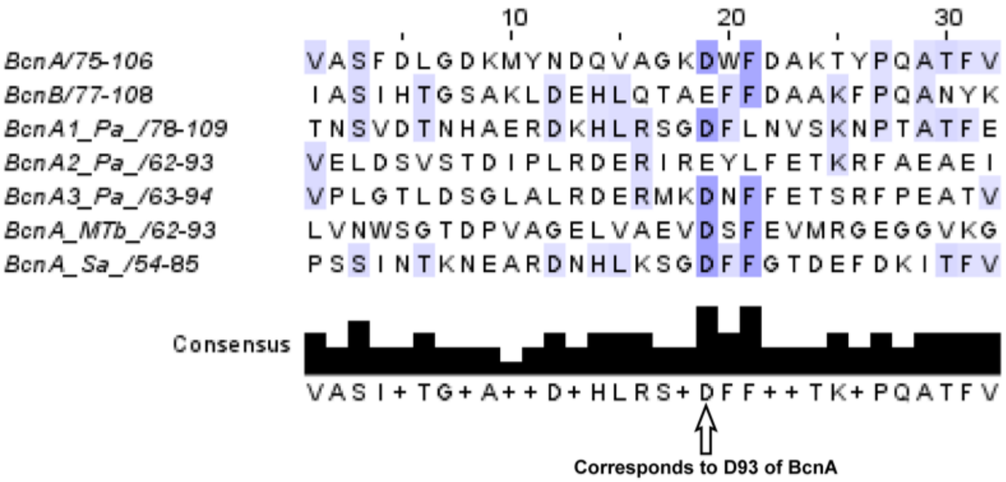

Fig. S5. Nile red binding affinity of *B. cenocepacia* BcnA site-directed mutants compared to the wild type, n=4, 2 independent experiments; the smooth lines show the non-linear least square regression analysis of the binding isotherms;

one-way ANOVA (with overall $p<0.001$) Dunn's Multiple Comparison Test (A-B). (C) Consensus motif of BCN family of proteins. (D) Sequence alignment and consensus motif of BCN homologues used in this study.
