# Supplementary material for: Antibiotic Capture by Bacterial Lipocalins Uncovers an Extracellular Mechanism of Intrinsic Antibiotic Resistance
Source: mBio. 2017 Mar 14;8(2):e00225-17. doi: 10.1128/mBio.00225-17 (PMC5350466; doi:10.1128/mBio.00225-17)
Supplement: FIG S6 [file mbo002173230sf6.pdf]

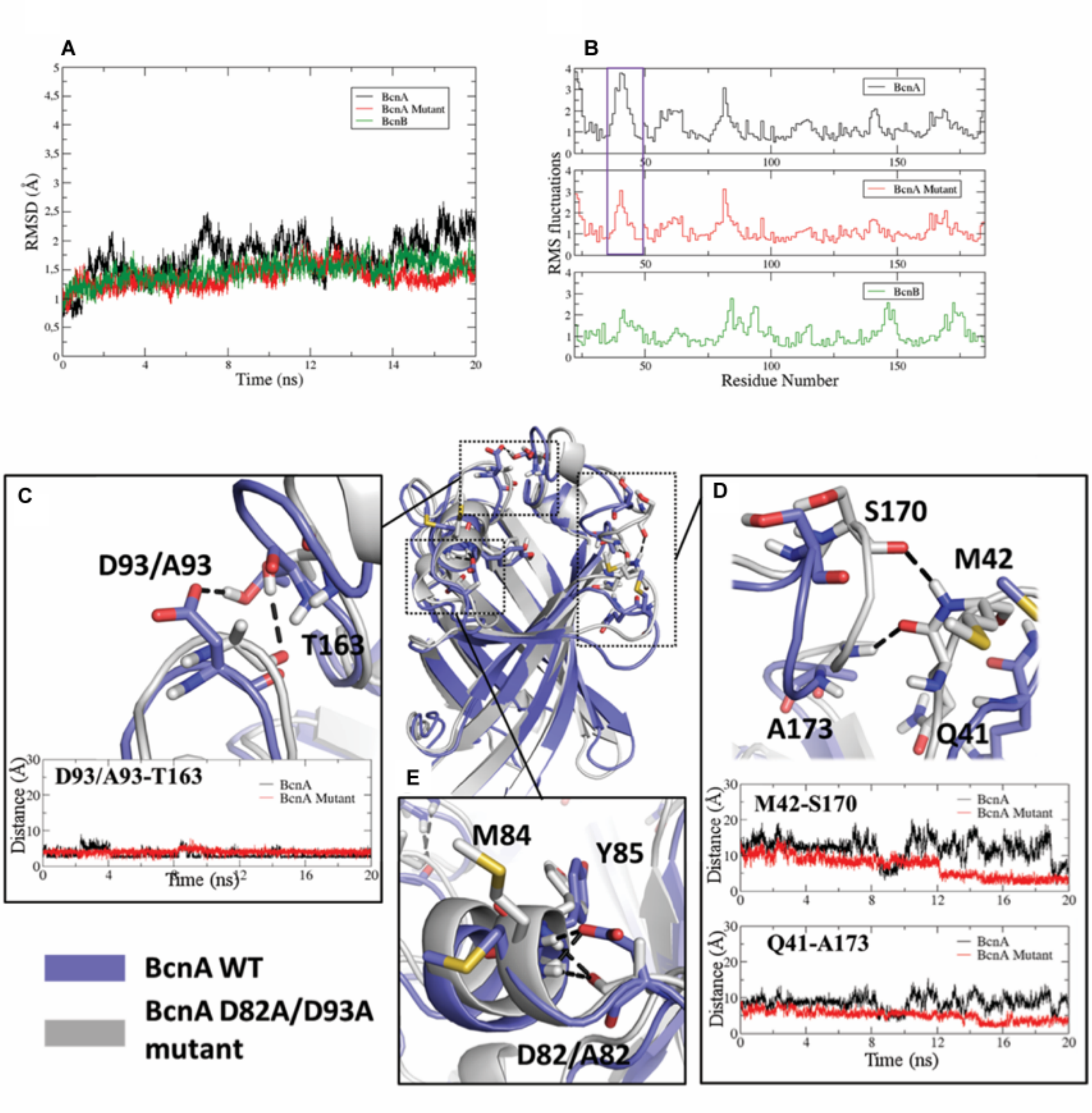

Fig. S6. (A-B) Overall MD simulation analysis of the BcnA, BcnA D82A-D93A mutant and BcnB structures. (A) Plot showing the RMSD of the position of the α-carbons of BcnA (black), BcnA D82A-D93A mutant (red) and BcnB (green) during the 20ns MD simulation. (B) Plot showing the RMS fluctuations of the position of all atoms during the 20ns MD simulation. The V38-E47 loop marked in the purple box remarks the higher flexibility observed in the case of the BcnA WT (black) compared to the BcnA D82A-D93A mutant (in red, lower fluctuations).

(C-E) Superimposition of the BcnA wild type (WT, indigo) and BcnA D82A/D93A mutant (grey) minimized average structures from the last 5ns of MD simulation. (C) Interaction between D93/A93 and T163 respectively, along with a plot representing the interaction distance between the D93 carboxylate oxygen and T163 side chain OH group in the BcnA WT, and the A93 carbonyl oxygen and T163 side chain OH group in the BcnA D82A/D93A mutant (marked with black dotted lines) along the MD simulation time. (D) Hydrogen bonds (marked with black dotted lines) between the M42 NH group and S170 CO group, and the Q41 CO group and A173 NH group (BcnA WT in indigo, and BcnA D82A/D93A mutant in grey), together with the plots of the corresponding interaction distances along the MD simulation time. (E) Detailed views of the hydrogen bonds established between the NH groups from M84 and Y85 and the D82 carboxylate oxygen in the BcnA WT, and the same NH groups from M84 and Y85 and the carbonyl oxygen from A82 in the BcnA D82A/D93A mutant (marked with black dotted lines).
