# Supplementary material for: Antibiotic Capture by Bacterial Lipocalins Uncovers an Extracellular Mechanism of Intrinsic Antibiotic Resistance
Source: mBio. 2017 Mar 14;8(2):e00225-17. doi: 10.1128/mBio.00225-17 (PMC5350466; doi:10.1128/mBio.00225-17)
Supplement: FIG S7 [file mbo002173230sf7.pdf]

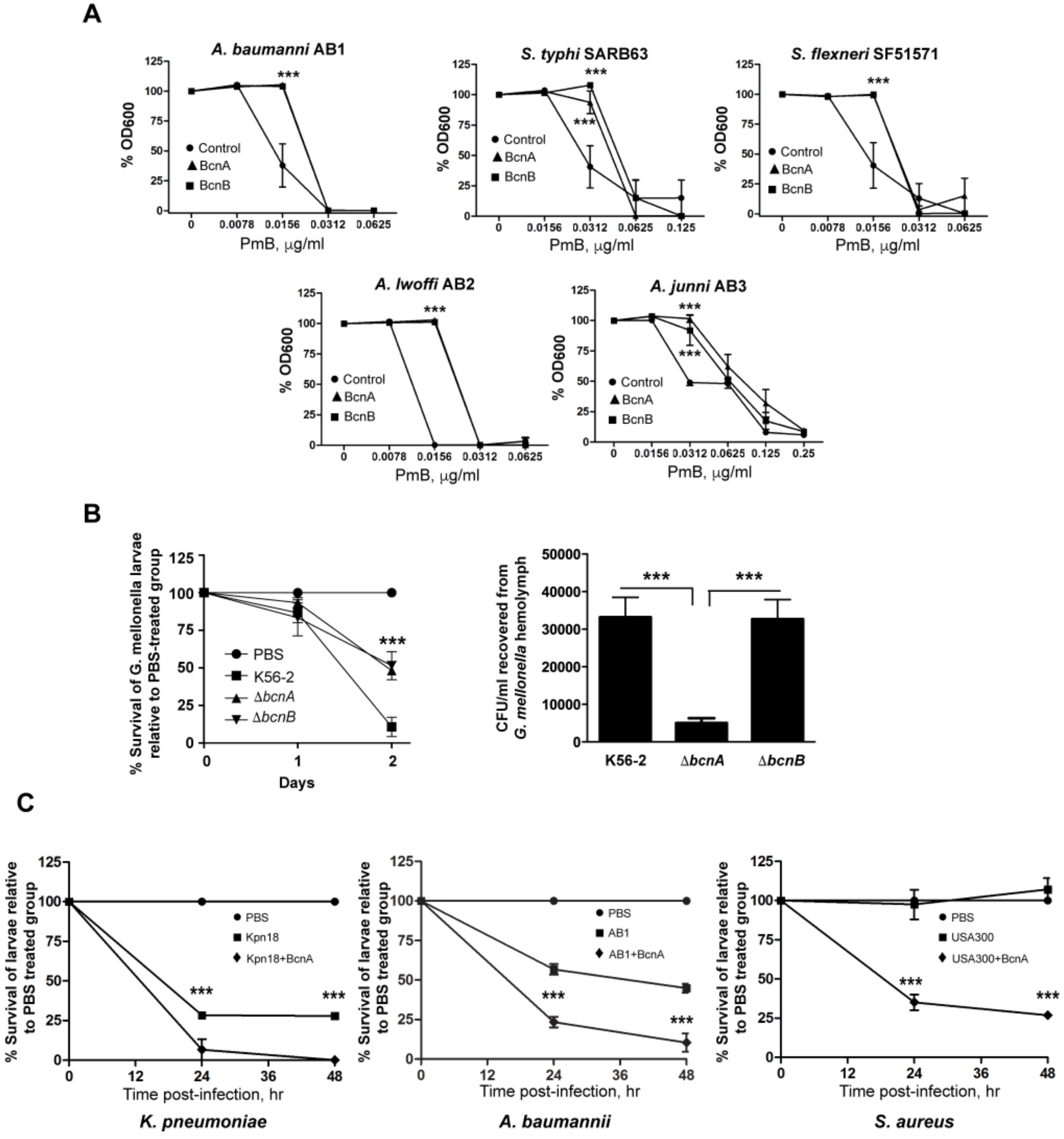

Fig. S7. Effects of *B. cenocepacia* BCNs on bacterial species *in vitro* and *in vivo*. (A) *in vitro* protection assays against PmB with 1.5 µM of BcnA or BcnB on *A. baumannii* AB1 (n=6 from 2 independent experiments), *Salmonella typhi* SARB63 (n=7 from 3 independent experiments), *Shigella flexneri* SF51571 (n=7 from 3 independent experiments), *Acinetobacter lwoffi* AB2 (n=5 from 2 independent experiments), and *Acinetobacter junii* AB3 (n=5 from 2 independent experiments). Mean ±SEM.* $p<0.05$, ** $p<0.01$ and *** $p<0.001$ determined by 2-way ANOVA (with overall $p<0.001$for AB1, AB2, AB3 and SF51571, $p=0.001$ for SARB63) and Bonferroni post-hoc tests. (B) Survival of Δ*bcnA* and Δ*bcnB* in *Galleria mellonella* larvae over a 48-h infection. 10 larvae per group; the results are obtained from 3 independent experiments and shown as mean of % larval survival in each experiment ±SEM. *** $p<0.001$ determined by 2-way ANOVA (with overall $p<0.001$) and Bonferroni post-hoc tests. At the chosen sample size (n), the actual power of the assay to detect statistically significant effects at significance level (alpha) of 0.05, two-tailed is 90-95%. The right graph shows the bacterial recovery from hemolymph at 200 min postinfection; n=10 from 2 independent experiments shown as mean ±SEM. ***

$p<0.001$ from one-way ANOVA test (overall $p<0.001$) and Bonferroni's post-hoc test. At the chosen sample size (n), the actual power of the assay to detect statistically significant effects at significance level (alpha) of 0.05, two-tailed is >99%. * $p<0.05$, *** $p<0.001$. (C) *in vivo* protection assay using *Galleria mellonella* infections. Each larva was injected with 10 μl of suspensions of different bacteria in PBS with or without BcnA. The survival was monitored over time and compared to control group injected with sterile PBS. Each group included 10 larvae. Data are from 3 independent experiments. * $p<0.05$, ** $p<0.01$ and *** $p<0.001$ determined by 2-way ANOVA (with overall $p<0.001$) and Bonferroni post-hoc tests compared to the respective infection control.
