# Supplementary material for: Antibiotic Capture by Bacterial Lipocalins Uncovers an Extracellular Mechanism of Intrinsic Antibiotic Resistance
Source: mBio. 2017 Mar 14;8(2):e00225-17. doi: 10.1128/mBio.00225-17 (PMC5350466; doi:10.1128/mBio.00225-17)
Supplement: TABLE S2 [file mbo002173230st2.pdf]

Table S2. Strains and Plasmids

Strain or plasmid	Relevant characteristics[a]	Source and/or reference
**Strains**		
Burkholderia cenocepacia		
K56-2	ET12 clone related to J2315, CF clinical Isolate	[b]BCRRC, (1)
OME19	K56-2 pSCrhaB2; Tp^R	(2)
OME37	K56-2 pOE12; BCAL3310 with C-terminus FLAG tag; Tet^R	This study
OME40	K56-2 pOE13; BCAL3311 with C-terminus FLAG tag; Tet^R	This study
OME60	K56-2, $P_{BCAL3310}$::pGSVTp-*luxCDABE*; Tp^R	This study
OME61	K56-2, $P_{BCAL3312\text{-}3311}$::pGSVTp-*luxCDABE*; Tp^R	This study
OME62	K56-2, ΔBCAL3311	This study
OME65	K56-2, ΔBCAL3310	This study
OME66	K56-2 pDA17; Tet^R	This study
OME71	OME62 pSCrhaB2; Tp^R	This study
OME72	OME62 pOE33 (BCAL3311); Tp^R	This study
OME73	OME62 pOE34 (PA0423); Tp^R	This study
OME74	OME62 pOE35 (PA4340); Tp^R	This study
OME75	OME62 pOE36 (PA4345); Tp^R	This study
OME76	OME62 pOE37 (Rv1890c); Tp^R	This study
OME77	OME62 pOE38 (SAUSA300_2620); Tp^R	This study
Escherichia coli		
DH5α	F^-ϕ80*lacZ* M15 *endA1 recA1 supE44 hsdR*17(r_K^- m_K^+)*deoR thi-1 nupG supE44 gyrA96relA1* Δ(*lacZYA-argF*)*U169, λ–*	Laboratory stock
GT115	F^- *mcrA*Δ(*mrr-hsdRMS-mcrBC*) ϕ80Δ*lacZ*ΔM15 Δ*lacX74 recA1 rpsL* (StrA) *endA1*Δ*dcm uidA*(ΔMluI)::*pir-116* Δ*sbcC-sbcD*	Invivogen, San Diego, CA
BL21	F^-*dcm ompT hsdS*($r_B^-$$m_B^-$) *gal*	Novagen
Pseudomonas aeruginosa		
PAO1	Non-CF clinical isolate	(3)
Q502	CF clinical isolate	(4)
Salmonella typhi		
SARB63		(5)
Shigella flexneri		
SF51571	Serotype 1a, antigenic formula 1:4	Laboratory stock
***Acinetobacter* species**		
A. baumannii (AB1)	Clinical isolate	LHSC[c]
A. lwoffi (AB2)	Clinical isolate	LHSC[c]
A. junii (AB3)	Clinical isolate	LHSC[c]

Klebsiella pneumoniae		
Kpn18	Clinical isolate	Laboratory stock
Staphylococcus aureus		
USA300	Community acquired MRSA	Martin McGavin
**Plasmids**		
pRK2013	ori_{colE1}, RK2 derivative, Kan^R, *mob*$^+$, *tra*$^+$	(6)
pGSVTp-*lux*	Mobilizable suicide vector containing lux operon, derivative from pGSV3-*lux*(7); OriT; Tp^R	(8)
pSCrhaB2	ori_{pBBR1}*rhaR*, *rhaS*, $P_{rhaB}$$Tp^R$*mob*$^+$	(9)
pDAI-SceI-SacB	ori_{pBBR1}, Tet^R, P_{dhfr}, *mob*$^+$, expressing I-SceI, SacB	(10)
pGPI-SceI	ori_{R6K}, ΩTp^R , *mob*$^+$, including an I-SceI restriction site	(11)
pDA17	ori_{pBBR1}, Tet^R, mob$^+$, P_{dhfr}, FLAG epitope	D. Aubert, unpublished
pOE12	pDA17, BCAL3310, C-terminus FLAG, Tet^R	This study
pOE13	pDA17, BCAL3311, C-terminus FLAG, Tet^R	This study
pOE15	BCAL3310 without signal peptide encoding sequence cloned in pET28a(+)	(2)
pOE16	BCAL3311 without signal peptide encoding sequence cloned in pET28a(+)	(2)
pOE22	$P_{BCAL3310}$::*luxCDABE* transcriptional fusion in pGSVTp-*lux*, Tp^R	This study
pOE23	$P_{BCAL3312\text{-}3311}$::*luxCDABE* transcriptional fusion in pGSVTp-*lux*, Tp^R	This study
pOE25	pGPI-SceI with fragments flanking BCAL3310, Tp^R	This study
pOE26	pGPI-SceI with fragments flanking BCAL3311, Tp^R	This study
pOE33	pSCrhaB2, BCAL3311, Tp^R	This study
pOE34	pSCrhaB2, PA0423, Tp^R	This study
pOE35	pSCrhaB2, PA4340, Tp^R	This study
pOE36	pSCrhaB2, PA4345, Tp^R	This study
pOE37	pSCrhaB2, Rv1890c, Tp^R	This study
pOE38	pSCrhaB2, SAUSA300_2620, Tp^R	This study
pOE39	pOE16, D82A	This study
pOE41	pOE16, D93A	This study
pOE46	pOE39, D93A	This study

[a]Tp^R, trimethoprim resistance, Kan^R, kanamycin resistance, Tet^R, tetracycline resistance.
[b]BCRRC, *B. cepacia* Research and Referral Repository for Canadian CF Clinics.
[c]LHSC, London Health Science Centre, London, Ontario, Canada.

TABLE REFERENCES

1. **Mahenthiralingam E, Coenye T, Chung JW, Speert DP, Govan JR, Taylor P, Vandamme P.** 2000. Diagnostically and experimentally useful panel of strains from the *Burkholderia cepacia* complex. J. Clin. Microbiol. **38:**910-913.

2. **El-Halfawy OM, Valvano MA.** 2013. Chemical communication of antibiotic resistance by a highly resistant subpopulation of bacterial cells. PLoS One **8:**e68874.

3. **Holloway BW.** 1955. Genetic recombination in *Pseudomonas aeruginosa*. J Gen Microbiol **13:**572-581.

4. **Camper N, Glasgow AM, Osbourn M, Quinn DJ, Small DM, McLean DT, Lundy FT, Elborn JS, McNally P, Ingram RJ, Weldon S, Taggart CC.** 2016. A secretory leukocyte protease inhibitor variant with improved activity against lung infection. Mucosal Immunol **9:**669-676.

5. **Boyd EF, Wang FS, Beltran P, Plock SA, Nelson K, Selander RK.** 1993. *Salmonella* reference collection B (SARB): strains of 37 serovars of subspecies I. J Gen Microbiol **139 Pt 6:**1125-1132.

6. **Figurski DH, Helinski DR.** 1979. Replication of an origin-containing derivative of plasmid RK2 dependent on a plasmid function provided in *trans*. Proc Natl Acad Sci USA **76:**1648-1652.

7. **Moore RA, Reckseidler-Zenteno S, Kim H, Nierman W, Yu Y, Tuanyok A, Warawa J, DeShazer D, Woods DE.** 2004. Contribution of gene loss to the pathogenic evolution of *Burkholderia pseudomallei* and *Burkholderia mallei*. Infect Immun **72:**4172-4187.

8. **Bernier SP, Nguyen DT, Sokol PA.** 2008. A LysR-type transcriptional regulator in *Burkholderia cenocepacia* influences colony morphology and virulence. Infect Immun **76:**38-47.

9. **Cardona ST, Valvano MA.** 2005. An expression vector containing a rhamnose-inducible promoter provides tightly regulated gene expression in *Burkholderia cenocepacia*. Plasmid **54:**219-228.

10. **Hamad MA, Skeldon AM, Valvano MA.** 2010. Construction of aminoglycoside-sensitive *Burkholderia cenocepacia* strains for use in studies of intracellular bacteria with the gentamicin protection assay. Appl Environ Microbiol **76:**3170-3176.

11. **Flannagan RS, Linn T, Valvano MA.** 2008. A system for the construction of targeted unmarked gene deletions in the genus *Burkholderia*. Environ. Microbiol. **10:**1652-1660.
